# Hemoadsorption Improves Survival of Rats Exposed to an Acutely Lethal Dose of Aflatoxin B_1_

**DOI:** 10.1038/s41598-020-57727-y

**Published:** 2020-01-21

**Authors:** Karl-Gustav Ruggeberg, Pamela O’Sullivan, Timothy J. Kovacs, Kathryn Dawson, Vincent J. Capponi, Phillip P. Chan, Thomas D. Golobish, Maryann C. Gruda

**Affiliations:** CytoSorbents Medical, Monmouth Junction, NJ, United States

**Keywords:** Cytokines, Proteomics, Biomarkers, Drug development, Preclinical research

## Abstract

Mycotoxins, such as aflatoxin B_1_ (AFB_1_), pose a serious threat as biological weapons due to their high toxicity, environmental stability, easy accessibility and lack of effective therapeutics. This study investigated if blood purification therapy with CytoSorb (CS) porous polymer beads could improve survival after a lethal aflatoxin dose (LD_90_). The effective treatment window and potential therapeutic mechanisms were also investigated. Sprague Dawley rats received a lethal dose of AFB_1_ (0.5–1.0 mg/kg) intravenously and hemoperfusion with a CS or Control device was initiated immediately, or after 30, 90, or 240-minute delays and conducted for 4 hours. The CS device removes AFB_1_ from circulation and significantly improves survival when initiated within 90 minutes of toxin administration. Treated subjects exhibited improved liver morphology and health scores. Changes in the levels of cytokines, leukocytes and platelets indicate a moderately-severe inflammatory response to acute toxin exposure. Quantitative proteomic analysis showed significant changes in the level of a broad spectrum of plasma proteins including serine protease/endopeptidase inhibitors, coagulation factors, complement proteins, carbonic anhydrases, and redox enzymes that ostensibly contribute to the therapeutic effect. Together, these results suggest that hemoadsorption with CS could be a viable countermeasure against acute mycotoxin exposure.

## Introduction

Aflatoxins are toxic secondary fungal metabolites (mycotoxins) produced by fungus from the genus *Aspergillus* that cause severe acute reactions that can be lethal. *Aspergillus* species are important human pathogens and the toxic metabolites appear to act as virulence factors to suppress the immune system in invasive aspergillosis^1^. Aflatoxins cause damage to the liver resulting in hemorrhagic liver necrosis, steatosis, bile duct proliferation and subsequent organ failure and have been detected in pulmonary lesions of immune-compromised patients with systemic aspergillosis^2^. Aflatoxin B_1_ (AFB_1_), the most potent toxin of the 14 naturally occurring aflatoxin variants, is extremely cytotoxic, genotoxic, and carcinogenic^3,4^. In the liver, cytochrome P450-modified AFB_1_ forms DNA adducts that lead to impaired cellular function, carcinogenesis and/or cell death and organ failure^5,6^.

Acute aflatoxin poisoning from mold contaminated foods has been linked with numerous deaths in several instances^7,8^. Importantly, mycotoxins pose a serious threat as potential biowarfare agents due to their inherent stability and ease of manufacture. The toxins can be readily weaponized into aerosol form and dispersed over a wide area to elicit mass casualties through both inhalation and dermal exposure^9^. There have been several reported incidents of use of mycotoxins as bioweapons in Southeast Asia and the Gulf States^10,11^. Early symptoms of mycotoxin exposure in bio-warfare manifest rapidly in minutes to hours and can include burning, pain, wheezing, nausea, vomiting, tearing, weakness, bleeding and a host of other symptoms depending on the route of exposure making preparedness a critical element of any medical countermeasure. Extracorporeal removal methods, in conjunction with supportive care, have been employed to treat victims of acute intoxication with varying results^12,13^. Of note, a successful outcome was reported in a case of acute aflatoxicosis with fulminant hepatic failure and rhabdomyolysis case treated with hemodiafiltration^14^. Also, a case of amanita phalloides-induced liver failure was successfully treated with the Molecular Adsorbent Recirculating System (MARS)^13^. Nonetheless, the optimal approach is often unclear given the limited reports and the rapid distribution of many mycotoxins relative to medical presentation^15^.

Mycotoxin-induced inflammation and necrosis releases intracellular proteins, called damage associated molecular pattern proteins (DAMPs), that cause further tissue damage. A recent study demonstrated the rapid removal of AFB_1_ and T-2 toxin, as well as various DAMPs and cytokines from blood by CytoSorb^®^ (CS; CytoSorbents Corporation, Monmouth Jct., NJ) porous polymer beads^16^, suggesting that hemoperfusion with CS could be useful in the treatment of acute mycotoxin exposure, such as might be predicted in a bioterrorist attack. In addition, intra-operative use of the device has been reported to reduce bleeding complications in patients who present for emergency cardiac surgery by the removal of the coagulation-active substances rivaroxaban and ticagrelor^17^. As such, it was of interest to evaluate if CS treatment could effectively mitigate the toxicity of an acutely lethal dose of AFB_1_ with systemic administration. Specifically, these studies were designed to determine if hemoperfusion with CS polymer beads could demonstrate *in vivo* AFB_1_ removal from circulation and ultimately improve survival of rats exposed to a lethal (LD_90_) dose of AFB_1_, to identify the effective treatment window through delayed hemoperfusion following AFB_1_ exposure, and lastly, to elucidate the impact of the hemoadsorption treatment on the plasma proteome.

## Results

### Aflatoxin in circulation

AFB_1_ concentration was measured in circulating plasma collected from the rats immediately after toxin injection and at various time points during and after hemoperfusion with Control and CS devices. Initial systemic AFB_1_ levels (T0) were not significantly different between Control and CS-treated groups in any of the treatment groups. Overall levels in the immediate, 30-minute, 90-minute, and 4-hour delayed treatment studies were 1464 ± 580 ng/mL, 1671 ± 743 ng/mL, 1579 ± 426 ng/mL, and 2703 ± 267 ng/mL, respectively.

**Figure 1:**
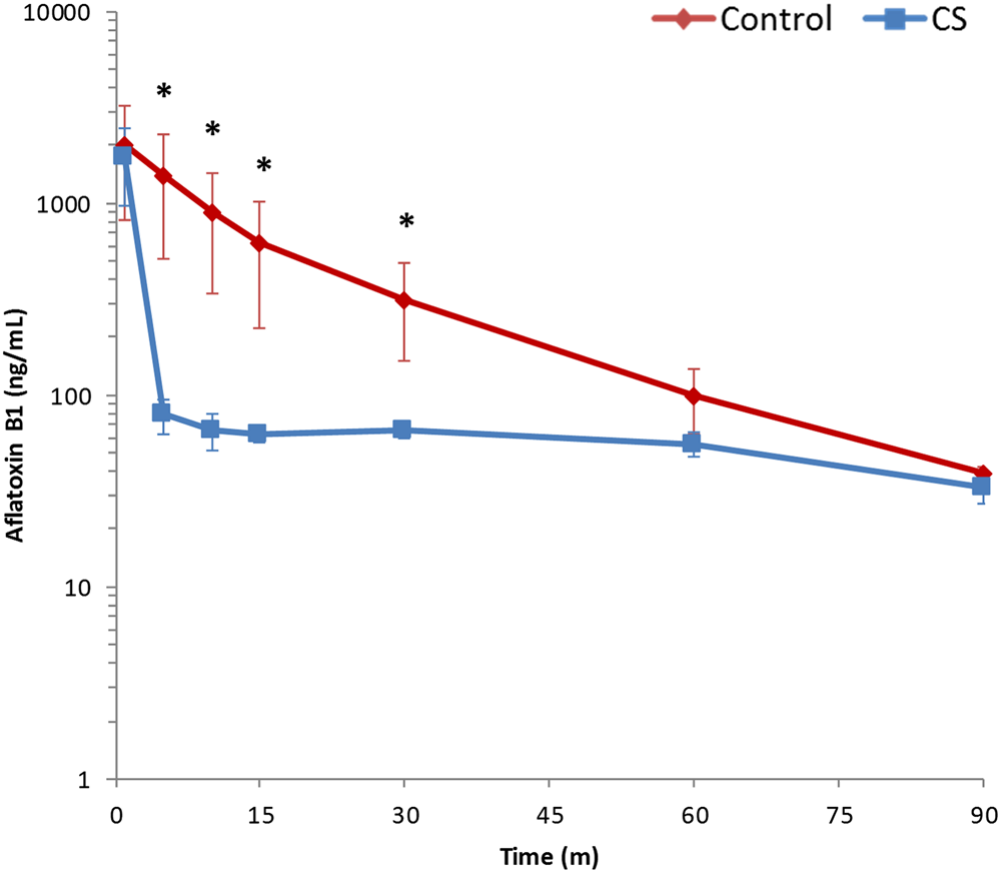
Circulating AFB_1_ Levels During Hemoperfusion. AFB_1_ levels measured by ELISA from plasma collected during hemoperfusion immediately after AFB_1_ IV administration (0.5 mg/kg). Hemoperfusion used either an empty (Control) or a CS device. Mean ± SD. n = 4 for both groups, *P < 0.05.

The CS device directly removed AFB_1_ from the blood stream as the clearance rate was significantly faster in the CS-treated animals than the natural clearance seen in the Control group (Fig. 1). Five minutes after AFB_1_ administration, 63.5% of the initial circulating AFB_1_ remained in the blood of the Control group, whereas just 5.2% was detectable in that of the CS-treated group. During the later time points, aflatoxin levels post-CS device remained low (under 5% of initial levels). This contrasts with the aflatoxin levels in the Control group, which gradually decreased from 64% at 5 minutes to 17% at 30 minutes. Circulating aflatoxin concentrations were significantly lower in the CS-treated rats versus those of the Control group at 5, 10, 15, and 30 minutes post dosing (P < 0.03; Fig. 1). At 90 minutes, aflatoxin levels were almost identical between the two groups, dropping to 3.6% and 2.4% of initial in the Control and CS-treated groups, respectively (Fig. 1).

The 30-minute delay study had similar average initial AFB_1_ levels between Control and CS-treated groups (Supplementary Fig. 1A). At 1.5 hours post injection, AFB_1_ levels dropped 62-fold to 25.1 ± 6.4 ng/mL in the Control group and 149-fold to 11.9 ± 2.9 ng/mL in the CS-treated group, a statistically significant difference (P < 0.0001). By 4.5 hours post injection, AFB_1_ levels were essentially identical between the Control and CS-treated groups at 3.3 ng/mL (P = 0.97).

At the start of the 90-minute delayed hemoperfusion, AFB_1_ levels had decreased by >99% to 6.8 ± 1.4 ng/mL in the Control group and to 6.6 ± 2.4 ng/mL in the CS-treated group; (P = 0.89; Supplementary Fig. 1B). By the end of the hemoperfusion session at 5.5 hours, toxin levels had decreased further to 1.2 ± 0.4 ng/mL and 1.0 ± 0.4 ng/mL in the Control and CS-treated group, respectively (P = 0.52). After 24 hours, circulating toxin levels had decreased by 99.98% to 0.3 ± 0.1 ng/mL in both groups (P = 0.85).

With the 4-hour delay, blood AFB_1_ levels were <0.1% of initial dose levels at 1.9 ± 0.7 ng/mL and 1.4 ± 0.6 ng/mL in the Control and CS-treated group, respectively (P = 0.12; Supplementary Fig. 1C) and decreased to 0.7 ± 0.3 ng/mL in the Control group and 0.5 ± 0.3 ng/mL in the CS-treated group by the end of the hemoperfusion treatment (P = 0.17). By 24 hours, toxin levels declined to less than 0.01% of initial dose in all groups.

### Survival

Preliminary dosing studies were used to adjust the IV dose of AFB_1_ to provide the targeted ~90% lethality within 3–4 days of dosing and ranged from 0.5–1.0 mg/kg among the immediate treatment, 30 and 90-min delayed treatment (0.5 mg/kg) and the 4-hour delayed treatment (1.0 mg/kg) studies.

**Figure 2:**
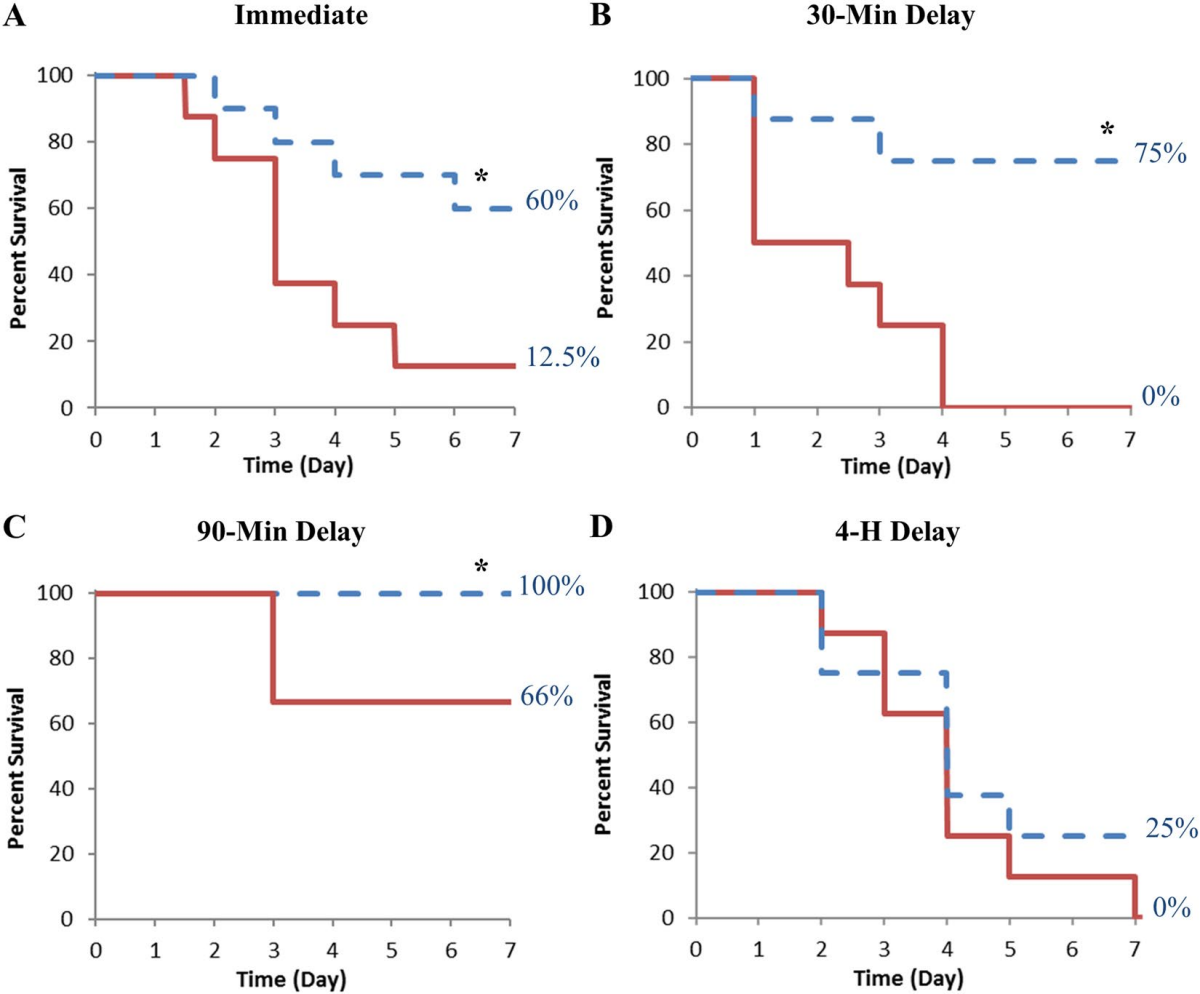
Survival Plots. Effect of hemoperfusion on survival following a lethal AFB_1_ dose. Rats were dosed with AFB_1_ IV and subjected to hemoperfusion with a Control or a CS device for 4 hours either (**A**) immediately after toxin injection (0.5 mg/kg AFB_1_); n = 8 and 10 for Control and CS groups, respectively, *P < 0.025, (**B**) after a 30-min delay (0.5 mg/kg AFB_1_); n = 8 for both groups, *P < 0.003, (**C**) after a 90-min delay (0.5 mg/kg AFB_1_); n = 12 and 11 for Control and CS groups, respectively, *P < 0.04, or (**D**) after a 4-h delay (1 mg/kg AFB_1_); n = 8 for both groups.

Animals treated with the CS device immediately after toxin administration lived significantly longer than the Control animals (P = 0.025). Animals in the Control group survived an average of 3.6 days, which confirms that the AFB_1_ dose was appropriate for this study. Sixty percent of the CS-treated rats survived until study conclusion on day 7 with the others dying between day 2 and 6 (Fig. 2A), whereas only 12.5% of the Control animals survived to study conclusion with the other animals dying between day 1 and 6 (Fig. 2A).

The survival advantage remained when treatment was delayed by 30 minutes (Fig. 2B) and 90 minutes (Fig. 2C). During the 7-day observation period after the 30-min delayed treatment, none of the Control rats survived to day 7, while 75% of the CS-treated rats survived (Fig. 2B). CS treatment 90 minutes after toxin dosing increased survival from 66% with the Control-treated rats (Fig. 2C) to 100% of the CS-treated rats surviving to the end of the study period (P = 0.04).

When treatment was delayed 4 hours, 2 of 8 CS-treated rats survived to the end of the study period, compared to 0 of 8 Control rats (Fig. 2D), a nonsignificant improvement of 25% with CS treatment (P = 0.32).

### Physical symptoms and weight loss

**Figure 3:**
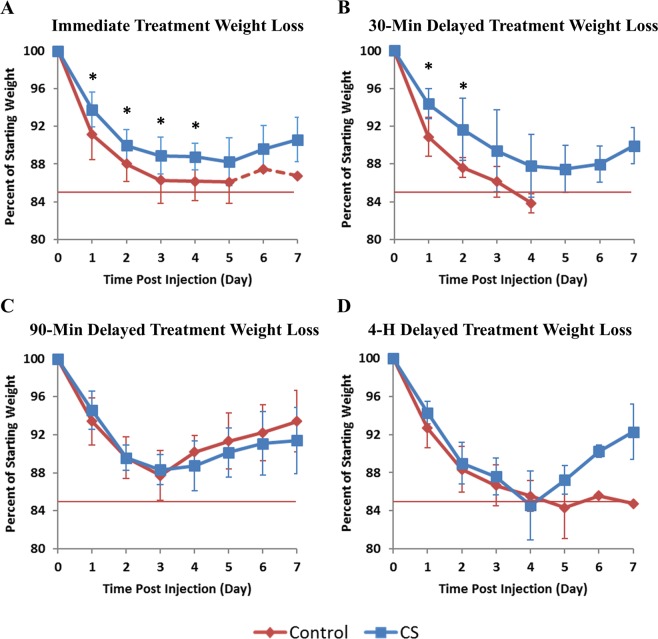
Body Weights. AFB_1_ -induced body weight loss. Rats received 0.5 mg/kg AFB_1_ dose (IV) (immediate, 30-min, and 90-min delayed treatment) or 1 mg/kg dose (4-h delayed treatment) and after the indicated delay, were connected to a hemoperfusion circuit containing either a Control or a CS device. Mean ± SD, T0 n ≥ 8. Dashed line represents sole surviving control rat, *P < 0.05. Red line indicates threshold for euthanasia.

Animals treated immediately with CS displayed a marked reduction in physical symptoms of aflatoxicosis compared to Controls; rats were more active, had less chromodacryorrhea, and more normal posture and grooming habits (Supplementary Table S2). As expected, AFB_1_ exposure caused a loss of body weight in both the Control and CS-treated animals; however, the animals that survived the first few days began to regain the lost weight (Fig. 3A). Control group animals lost significantly more body weight than CS-treated rats by day 1 post-injection: 8.9 ± 2.7% vs. 6.2 ± 1.9% (P = 0.02). By day 2, Control rats lost 12.0 ± 1.9% body weight; significantly more weight loss than that experienced by CS-treated rats – 10.0 ± 1.7% (P = 0.04). This trend continued through day 4, where the surviving Control rats had lost 13.8 ± 2.1% body weight compared to 11.2 ± 1.4% for CS-treated rats (P = 0.04). On the last day of survival, rats in the Control group had a significantly greater weight loss than CS-treated animals (P = 0.005).

The Control animals in the 30-minute delayed treatment group exhibited clear signs of AFB_1_ poisoning, including lethargy, chromodacryorrhea, rough coat, and hypothermia. These symptoms were largely absent from the CS-treated rats (Supplementary Table S2). Control group animals lost significantly more body weight than CS-treated rats by day 1 post-injection: 9.1 ± 2.0% vs. 5.6 ± 1.6% P = 0.002). By day 2, Control rats lost 12.4 ± 1.0% body weight; significantly more weight loss than that experienced by CS-treated rats – 8.3 ± 3.3% (P = 0.04) (Fig. 3B). By day 7, the surviving CS-treated rats had regained 20% of the weight lost during the study. Direct comparison of weight lost in CS-treated rats that received immediate treatment versus those that had a 30-minute delay of treatment showed no significant difference between the groups at any point during the study (P < 0.22).

Delaying treatment by 90 minutes also caused a reduction in chromodacryorrhea and improved grooming habits in CS-treated rats vs. Control rats (Supplementary Table S1). As expected, both Control and CS groups experienced significant weight loss (Fig. 3C). On day 1, Control rats lost 6.6% and CS rats lost 5.4% body weight, but there was no significant difference between the two groups (P = 0.71). On day 2, weight loss continued with both groups losing 10.4% body weight. The maximum average weight loss occurred on day 3 and was similar between Control and CS groups at 12.3% and 11.7%, respectively (P = 0.57). From day 4 through 7, both Control and CS-treated rats began to regain weight.

A 4-hour delay of treatment caused a reduction in toxin-mediated chromodacryorrhea and hypothermia but increased the incidence of lethargy in CS-treated vs. Control rats (Supplementary Table S2). Significant weight loss also occurred in this study, with Control and CS-treated groups losing similar amounts of weight. On day 1, Control rats lost 7.3% and CS-treated rats lost 5.7% body weight; however, there was no significant difference (P = 0.07). On day 2, rats continued to lose weight; Control rats lost 11.7% and CS-treated rats lost 11% (P = 0.58). Peak average weight loss occurred on day 4 and 5 for CS-treated rats (15.5%) and Control rats (15.7%), respectively. From day 4 through 7, the 2 surviving CS-treated rats were regaining weight (Fig. 3D).

### Liver morphology

**Figure 4:**
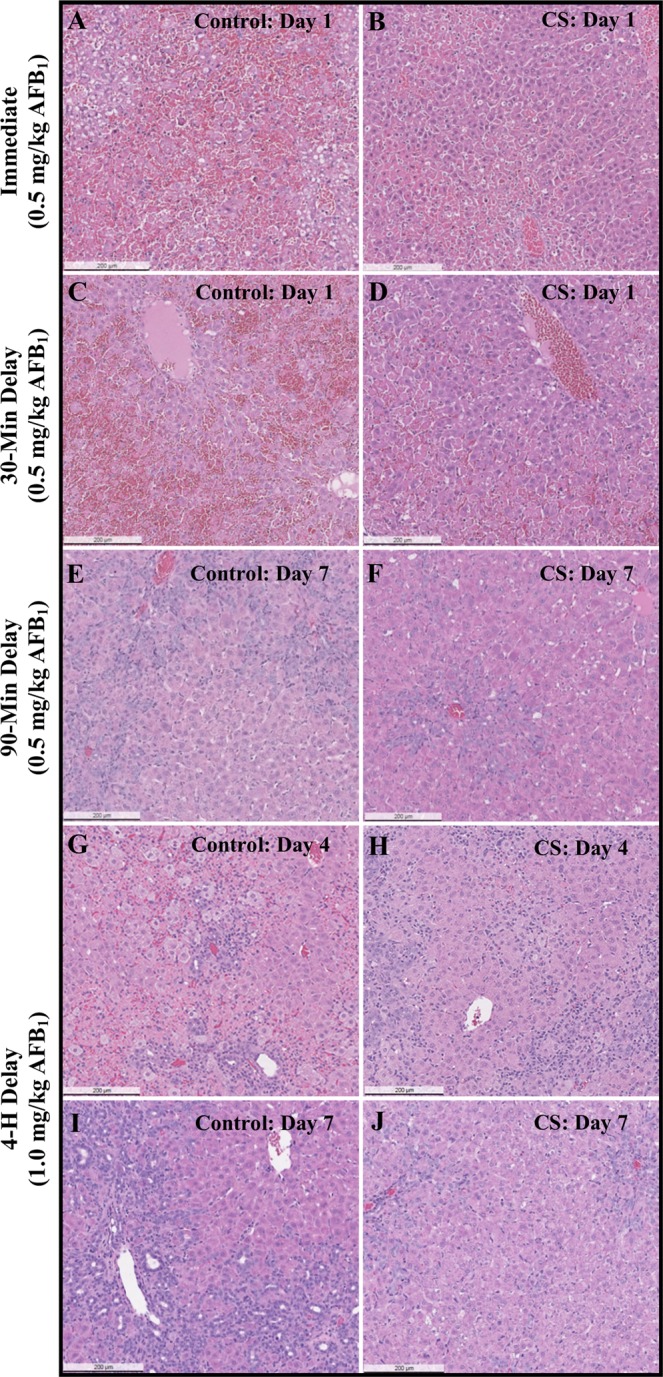
Liver Pathology. Liver pathology caused by acute AFB_1_ intoxication. H&E stained liver sections from rats injected with AFB_1_ IV and treated with either a Control or CS device as indicated. Areas of severe hemorrhage and hepatocyte necrosis are visible in tissues at day 1. (**A**–**D**) On day 3–4, leukocyte infiltration is present. (**G**,**H**) Biliary hyperplasia is evident in the day 7 toxin-exposed animals (**F**,**I**,**J**). 100X mag.

**Figure 5:**
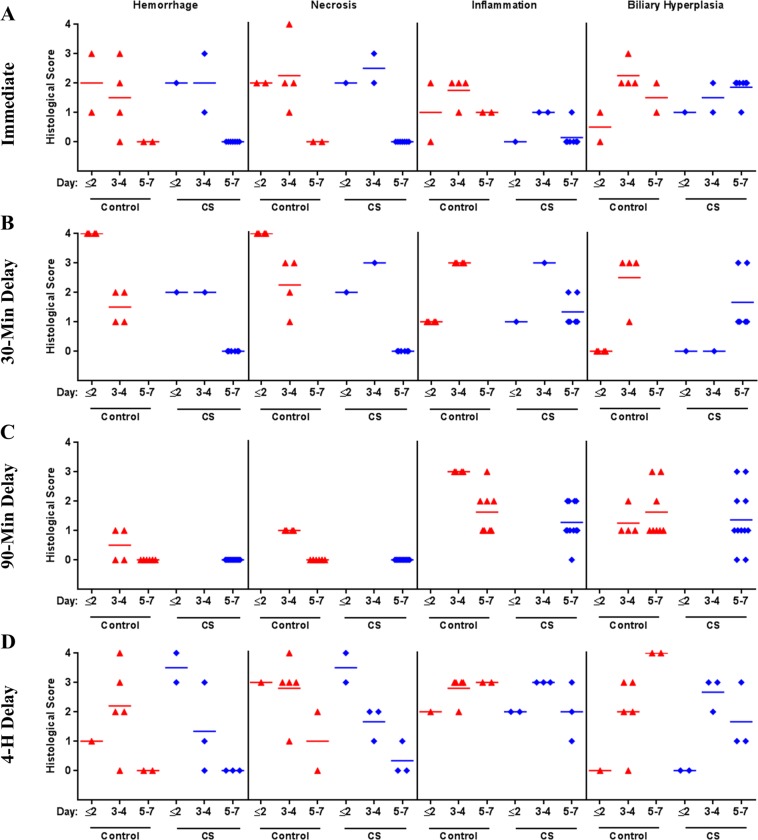
Histological Scores. Scores of liver sections from AFB_1_ injected animals. Treatment with either a Control or CS device began immediately (**A**), after a 30-min delay (**B**), a 90-min delay (**C**), or a 4-h delay. (**D**) Dose: 0.5 mg/kg AFB_1_ for immediate, 30-min, and 90-min delayed treatments; 1 mg/kg for 4-h delayed treatment. Key markers of toxin-induced damage: necrosis, hemorrhage, inflammation, and hyperplasia were scored on increasing severity from 0 through 4, with 0 representing normal healthy tissue.

No gross lesions were noted upon necropsy in any of the rats dosed with AFB_1_. Histological analysis of liver sections collected at death revealed a distinct difference in the degree of AFB_1_-induced liver damage between the Control and CS-treated rats. Rats that died during the first 3 days had large hemorrhagic areas throughout the parenchyma with biliary hyperplasia and hepatocyte apoptosis and little to no liver regeneration (Fig. 4A,B). Rat livers were scored for key markers of toxin-induced damage; Control rat livers exhibited greater necrosis, inflammation, and hemorrhage than those of CS-treated rats (Fig. 5A and Supplementary Table S3). Biliary hyperplasia was higher in Control rat livers that survived through day 4; however, CS-treated animals that survived through day 7 exhibited greater hyperplasia than Control rats (Fig. 5A and Supplementary Table S3). Surviving CS-treated rats had significant regeneration of the liver, reduced inflammation, and residual tissue fibrosis (Fig. 5A and Supplementary Table S3). Rank order analysis of severity of tissue damage showed that, with one exception, livers from CS-treated rats had less toxin-associated lesions than those of the Controls.

Delaying treatment for 30 minutes did not impact the therapeutic effects of CS treatment. Among animals that died on day 1, there was substantially more hemorrhaging and hepatocyte necrosis in livers of the Control group than in the CS-treated group (Figs. 4C,D, 5B and Supplementary Table S3). At day 7, the surviving CS-treated rats exhibited clear signs of hepatocyte regeneration in the parenchyma with modest inflammation; residual tissue fibrosis and biliary hyperplasia were also present (Fig. 5B and Supplementary Table S3).

Animals from the 90-minute delayed treatment study exhibited less overall liver damage, which agrees with the decreased mortality observed in the Control group. Control animals that died on day 3 had hemorrhaging and substantial leukocyte infiltration in the parenchyma (Fig. 5C and Supplementary Table S3). At day 7, liver regeneration had occurred in surviving Control and CS rats, with more inflammation present among Control animals. Again, moderate fibrosis and hyperplasia were evident around the portal triad (Figs. 4E,F, 5C and Supplementary Table S3).

The 4-hour delay of treatment animals experienced more severe liver damage than those of the 90-minute delay study, in congruence with the low survival outcome of both groups in this study. CS animals that died on day 2 had greater hemorrhage and necrosis than Control animals, but the difference was not significant. Control rats that died between day 3 and 4 had greater hemorrhage and necrosis, yet lower inflammation and hyperplasia than CS-treated animals; however, these differences were not significant (Figs. 4G,H, 5D and Supplementary Table S3). Animals that survived to day 5 to 7 had no detectable liver hemorrhage, minimal necrosis, substantial inflammation, and obvious biliary hyperplasia (Fig. 4I,J, 5D and Supplementary Table S3). The CS-treated animals appeared to have less severe lesions; however, differences were not significant.

**Figure 6:**
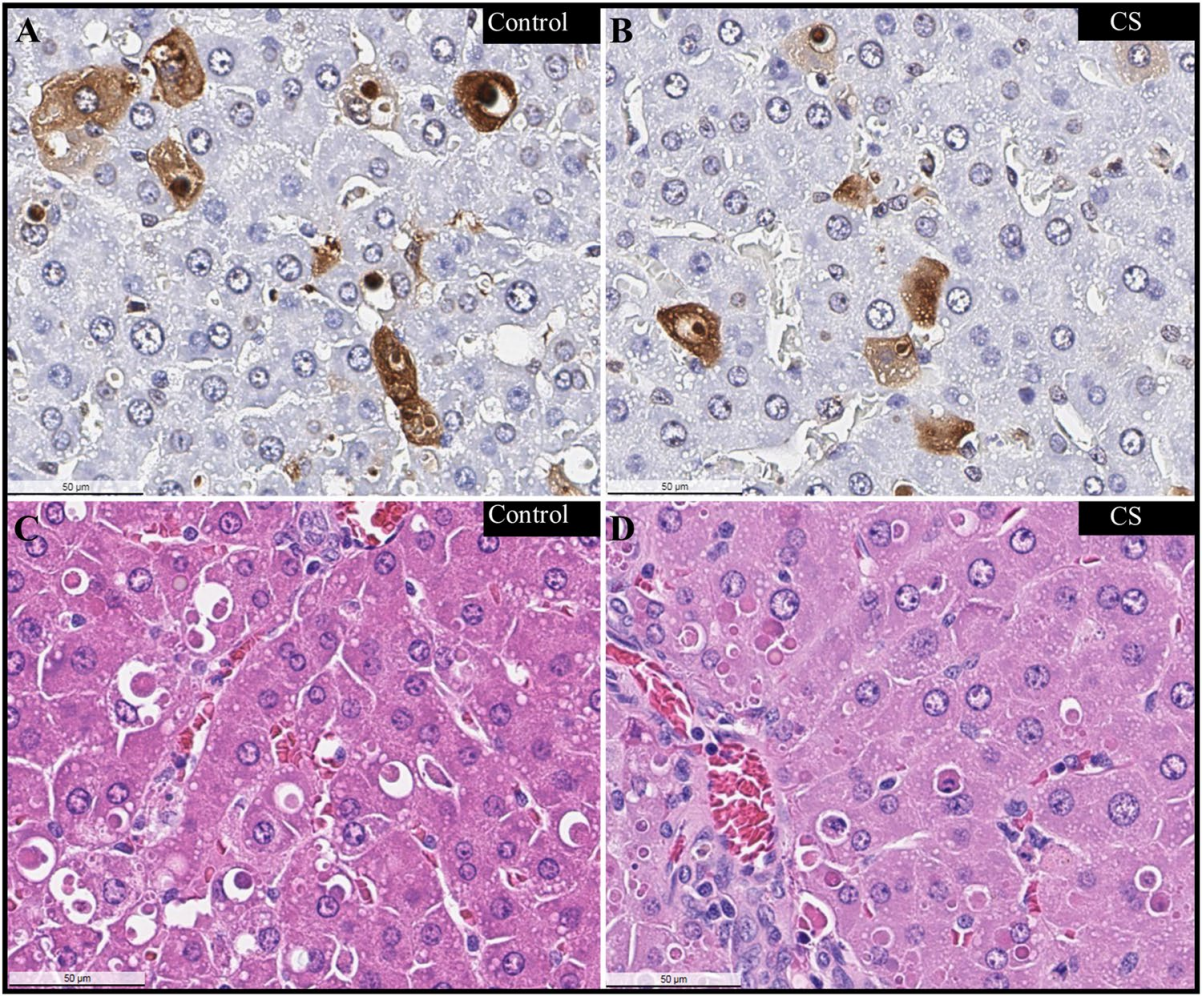
Hepatocyte Apoptosis. AFB_1_-induced hepatocyte apoptosis in rats treated for 4 hours with either a Control or CS device 90-minutes after AFB_1_ dosing (1.0 mg/kg) then sacrificed. Liver sections were TUNEL stained to visualize apoptotic cells (stained brown) (**A**,**B**) or H&E-stained for gross morphology (**C**,**D**). 400X mag.

Direct AFB_1_-induced hepatocyte apoptosis was assessed in rats that received 90-minute delayed treatment after a 1 mg/kg AFB_1_ dose using terminal deoxynucleotidyl transferase dUTP nick-end labeling (TUNEL). At 5.5 hours post-toxin dosing, there was substantial apoptosis visible in the parenchyma: 9.0 ± 2.4% and 9.3 ± 1.5% of liver cells in the Control and CS-treated rats were apoptotic, respectively, underscoring the rapid hepatotoxic effects of the AFB_1_ dose (Fig. 6).

### Circulating blood counts

Aflatoxicosis has been reported to cause lymphocytopenia and monocytopenia with increased neutrophil levels in certain species^18^, so changes in hematology were of interest in these studies. A complete blood count (CBC) was performed during the immediate and delayed treatment studies to analyze the direct AFB_1_ effects on the blood cell populations and any modulatory effects of CS treatment. Overall, AFB_1_ caused a 30–50% drop in white blood cell (WBC) numbers, which proceeded to rebound over the ensuing 24 hours, eventually reaching/surpassing baseline levels in all four studies. Except for immediate treatment, CS did not impact WBC counts. AFB_1_ had minimal effects on platelet and erythrocyte number, with the exception of the 30-min delayed treatment study, which showed a significant 46% decline in platelet numbers only in the Control group, and the 4-hour delayed treatment study, where erythrocyte number was significantly lower in the CS-treated group at 24 hours (Supplementary Table S4).

### Cytokine response

**Figure 7:**
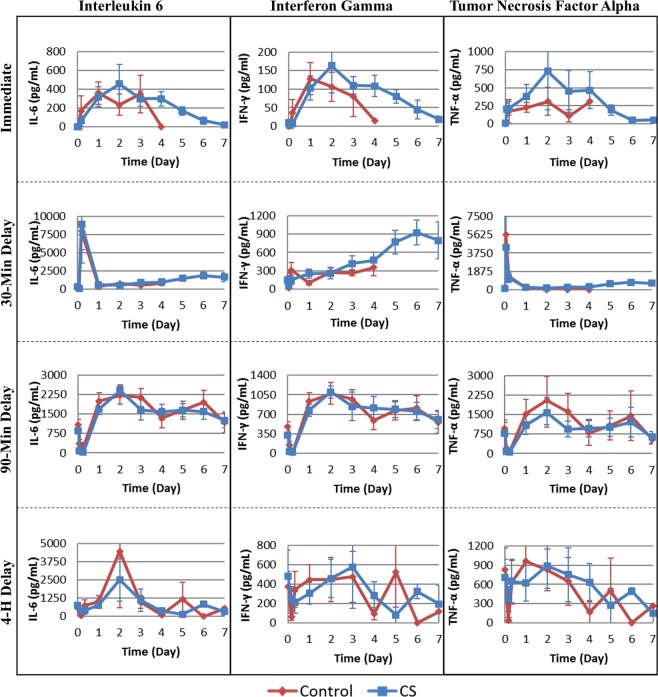
Cytokine Levels. Effect of AFB_1_ injection and subsequent hemoperfusion on levels of key circulating pro-inflammatory cytokines. AFB_1_ dose (mg/kg) was administered at time 0 with the hemoperfusion starting immediately (0.5 mg/kg), at 30 min (0.5 mg/kg), at 90 min (0.5 mg/kg), or at 4 hours (1.0 mg/kg). Mean ± SEM.

AFB_1_ poisoning has been reported to cause extensive physiological effects from cellular damage, which can stimulate a widespread inflammatory response. As such, the impact of AFB_1_ administration on circulating cytokine levels as well as the influence of hemoperfusion on the cytokine response was assessed by immunoassay. Pro-inflammatory cytokines IL-6, IFN-γ, and TNF-α were elevated above baseline, peaking hours to days after AFB_1_ injection in both Control and CS-treated groups of the immediate, 30-minute and 90-minute delayed treatment studies (Fig. 7). IL-6 and IFN-γ were significantly elevated above baseline in the immediate, 30-minute and 90-minute delayed treatment studies, while TNF-α was significantly increased in the 30-minute delayed treatment study (Supplementary Tables S4–6). These changes were not significant in the case of the 4-hour delayed treatment study. IL-1α, IL-1β, IL-2, IL-4, IL-5, IL-10, IL-12, IL-13, and GM-CSF levels were also elevated over the course of the 7 days following AFB_1_ injection. IL-1α, IL-2, IL-4, IL-5, IL-10, IL-12 were significantly elevated in the immediate, 30-minute, and 90-minute studies, IL-1β and IL-13 were significantly elevated in the 30-minute and 90-minute studies, and GM-CSF was significantly increased above baseline in the 90-minute study (Supplementary Figs. S2–4 and Tables S4–6).

### Modulation of protein levels

Since minimal AFB_1_ remains in circulation 90 minutes post-toxin dosing, identifying the role of direct adsorption or indirect removal of DAMPs and inflammatory proteins on the therapeutic mechanism responsible for the increased survival with CS treatment following AFB_1_ exposure (Fig. 2C) was of interest. Therefore, a comprehensive proteomic analysis of plasma samples from a 90-minute delayed treatment study following 1 mg/kg AFB_1_ dose was utilized to identify and quantify proteins involved in the toxin-mediated damage.

**Figure 8:**
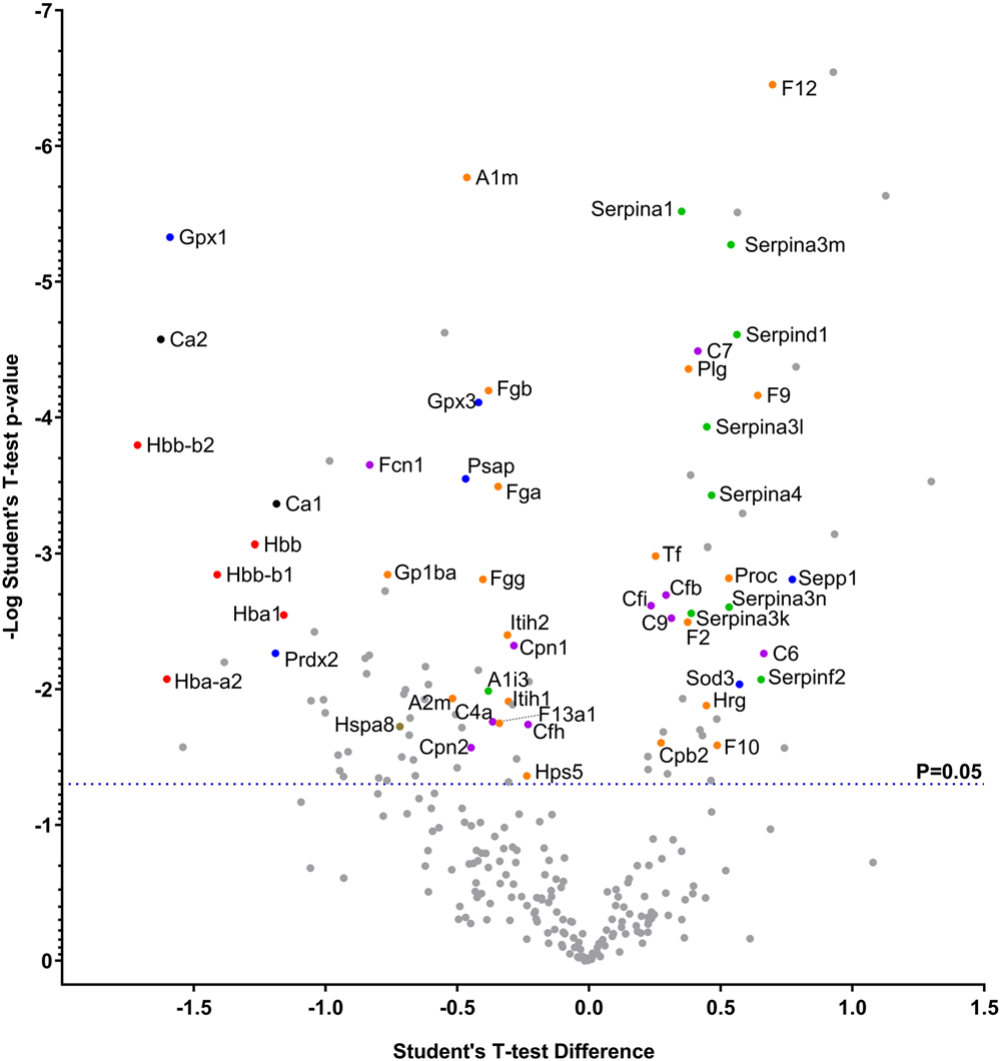
Proteomic Analysis. Effect of CS treatment on AFB_1_ (1.0 mg/kg)-induced changes in circulating protein abundance at 5.5 hours (n = 8). Key proteins that were significantly different between Control and CS treatment (P < 0.05) are color coded by function: red, hemoglobin; black, pH and fluid homeostasis; orange, coagulation; purple, complement; green, protease inhibitor; blue, ROS detoxification; brown, heat shock. Mean difference (Control-CS) vs. statistical P-value at 5.5 h post-toxin dose following 4 hours of hemoperfusion.

Over 300 unique proteins were unambiguously identified by liquid chromatography-mass spectrometry (LC-MS) proteomic analysis of the plasma samples. Examination of the samples 90 minutes following AFB_1_ administration, and prior to treatment, identified 34 proteins that were significantly altered in abundance (Supplementary Table S8) clustered around increases in complement proteins and serine protease inhibitors with decreased levels of hemoglobin subunits. Following hemoperfusion, at 5.5 hours post-toxin, 72 proteins were significantly altered from baseline in the Control animals, indicating further AFB_1_-induced changes (Supplementary Table S9). Serine protease inhibitors remained elevated with additional isoforms identified as well as significant increases in various coagulation factors. Hemoglobin subunits again were below baseline levels, as were a number of apolipoproteins. AFB_1_ induced significant changes in abundanc e of carbonic anhydrase 1 and 2 (CA1 and CA2) in the Control group after 1.5 hours with CA1 remaining significantly below baseline after 5.5 hours. AFB_1_ also impacted the abundance of two important redox enzymes, increasing superoxide dismutase 3 (SOD3) and decreasing peroxiredoxin-2 (PRDX2) levels at 1.5 hours. Examining the CS versus Control treatment effect (5.5 hours post-toxin), identified 109 proteins that were significantly different in abundance (Fig. 8 and Supplementary Table S10). Eight serine protease inhibitor isoforms were significantly decreased in the CS treated animals compared to Controls suggesting the importance of this group to the therapeutic effect. Levels of intrinsic coagulation factors and carboxypeptidase B2 (CPB2) are decreased in the CS treated animals compared to the Controls, while later stage common pathway factors remained elevated. Similarly, many complement proteins are decreased at 5.5 hours post-toxin in the CS compared to the Control animals where 8 different complement proteins were increased from baseline, suggesting another means of CS treatment may reduce toxin-mediated inflammation. CS treatment caused a significant increase in the abundance of both carbonic anhydrases when compared to the Control group at 5.5 hours post-toxin dosing. CS treatment resulted in increased PRDX2 and decreased SOD3 levels compared to those of the Control group. Additionally, treatment increased the level of prosaposin-isoform A (PSAP-1), glutathione peroxidase 1 (GPx-1), and glutathione peroxidase 3 (GPx-3).

## Discussion

These studies demonstrate that treatment with the CS hemadsorption device removes AFB_1_ from the blood stream and significantly improves survival after exposure to an acutely lethal dose of AFB_1_ when treatment is initiated within 90 minutes of administration. The reduced scale device used in this study is based on the 300 mL CytoSorb^®^ adsorber, a CE-Mark certified device approved for use as an extracorporeal cytokine adsorber. The indications for use have been expanded to include the adsorption of myoglobin^19^ and bilirubin^20^. The device has been shown to adsorb a number of small lipophilic molecules from blood *in vitro* including amitriptyline, diazepam, digoxin, quetiapine, and rivaroxaban^21^ as well as aflatoxin and T-2 toxin^16^, which prompted this investigation. Notably the device has been used alongside supportive care to treat intoxication by the serotonin and norepinephrine reuptake inhibitor, venlafaxine and the mushroom product psilocybin^22,23^, illustrating the translational use in treating acute toxin poisoning.

Treatment with the CS device remains just as effective in improving survival, liver morphology, and health scores when delayed by 30 or 90 minutes as when applied immediately after direct systemic administration of the toxin (Figs. 2–5; Supplementary Tables S2–3). The broad removal capabilities of the device suggest several possible, complementary mechanisms for the survival benefit observed with this acute mycotoxin poisoning model. Direct aflatoxin adsorption can readily explain at least part of the survival benefit observed when treatment is initiated within 30 minutes of AFB_1_ exposure as substantial levels of aflatoxin remain in circulation. However, the survival benefit extends beyond this time pointing to modulation of the plasma proteome as an additional therapeutic mechanism. CS has been shown to remove DAMPs^16^, including activated complement, indicating that the reduction of tissue damage associated and inflammatory mediators as a likely explanation for the survival benefit observed when treatment is initiated when nominal levels of toxin remain in circulation. A reduction in the primary and secondary release of DAMPs and inflammatory factors could be an additional mechanism to explain the treatment efficacy, beyond direct AFB_1_ adsorption. This is supported by the proteomic analysis that identified a broad spectrum of proteins from different biochemical pathways that were modulated by CS treatment. Five notable protein groups were influenced by the treatment: serine protease/endopeptidase inhibitors, coagulation factors, complement proteins, carbonic anhydrases, and redox enzymes (Fig. 8). The broad range of affected pathways is similar to proteomic surveys of acute-on-chronic liver failure, which causes sweeping changes in the plasma proteome^24,25^ and an *in vitro* model of mycotoxin-induced hepatocyte toxicity that found non-housekeeping pathway proteins to be affected most^26^.

AFB_1_ causes significant hemorrhage in the liver and intrinsic coagulation factors are fundamental to clot formation in response to vascular damage. However excessive clot formation can result in thrombosis, poor microvascular perfusion and systemic depletion of coagulation factors. A reduction in the level of several intrinsic coagulation factors and CPB2 could prevent excessive clotting and expedite existing clot fibrinolysis, possibly resulting in stabilization of hemostasis and protection of the endothelial layer. Furthermore, serine protease inhibitors help regulate proteolytic cascades that directly modulate coagulation, inflammation and immune responses, and tissue remodeling^27^ providing multiple potential beneficial mechanism(s) of action of their removal. The decrease in complement factors, which are vital components of the innate immune system that promote inflammation^28^, observed in the present study may reduce toxin-induced tissue necrosis and systemic inflammation as a potential therapeutic action of CS treatment.

Acutely toxic doses of AFB_1_ rapidly overwhelm endogenous redox enzyme-mediated detoxification, leading to widespread inflammation and release of DAMPS, amplifying the direct toxin effects^29–32^. Indeed, reactive aflatoxin metabolites can directly bind nucleic acids, disrupt protein synthesis and ultimately cause widespread cellular damage^7,32,33^. Consistent with prior reports^34,35^, elevated levels of TNF-α and IFN-γ were observed. Coupled with an excess of reactive oxygen species (ROS), TNF-α has been shown to cause hepatocyte apoptosis^36–38^. Although CS-treated rats had similarly elevated levels of TNF-α after AFB_1_ dosing as those of Control animals, there was markedly reduced liver damage (Fig. 5 and Supplementary Table S2), indicating an alternative to cytokine removal alone may explain the reduced tissue damage.

Carbonic anhydrases transport and remove dissolved carbon dioxide from the tissues and circulation; their depletion could result in decreased systemic pH as well as hyperammonemia, elevated lactate, metabolic acidosis and hypoglycemia. It is apparent that CS treatment abrogates the observed AFB_1_-mediated depletion of these enzymes, which could contribute to the overall therapeutic effect of the treatment. It is known that AFB_1_ detoxification causes elevated levels of ROS^39–41^. SOD directly converts superoxide into less-reactive oxygen species such as hydrogen peroxide and oxygen, whereas PRDX catalyzes the breakdown of hydrogen peroxide into water. While CS treatment moderately reduced the levels of SOD3, it caused a substantially larger increase in PRDX2. PRDX2 is a key component of the downstream ROS detoxification pathway^39–42^. Depending on the type of ROS present during CS treatment after AFB_1_ dosing, SOD3 may not be as critical as PRDX2 in preventing further tissue damage. Moreover, CS treatment also increased the level of GPx-1 and GPx-3, which directly catalyze the reduction of organic ROS and hydrogen peroxide by glutathione^43^. An increase in their protein abundance will likely result in decreased accumulation of ROS species, thereby protecting cells against further oxidative damage.

The changes observed in cytokines, WBC and platelet counts, are consistent with a moderately severe inflammatory response to acute toxin exposure. Furthermore, the rapid decline in WBCs after the single toxin dose parallels the effect seen in chronic AFB_1_ animal models^14,18^. However, unlike the consistently reduced WBC numbers observed with chronic toxin exposure, a single acute AFB_1_ dose causes a transient decline in WBCs followed by a rebound above baseline (Supplementary Table S3). This is supported by Marin 2002, who observed a biphasic response of decreasing and increasing WBC counts, with low and high toxin concentrations in piglets^44^. Immediate CS treatment may limit the subsequent toxin-induced increase of circulating WBCs; however, this effect is not observed when CS treatment is delayed by 30 or 90 minutes, limiting the generalizability of this observation.

Early hemoperfusion approaches to treat intoxication with modified charcoal have been reported to have undesirable effects on hemostasis and coagulation activity leading to their disuse^45^. In contrast, longer-term clinical studies (30- & 60-day) have established CS treatment to be safe and effective for a number of indications in critically ill patients^46^ easing general concerns about disturbances to physiologic homeostasis through untoward removal of beneficial substances, however close drug level monitoring is essential as some antibiotics and other drugs are known to be adsorbed^47^.

Overall, this study supports further investigation into the possibility of implementing CS treatment as a medical countermeasure against potential bio-warfare attacks with mycotoxins to lessen loss of life in military or civilian casualty situations if rapid deployment mechanisms are implemented. Consideration of such logistics is prudent, given the broad-spectrum nature of the device as a hemoadsorbent to address not only the initial toxin adsorption but the subsequent tissue damaging inflammatory response. This secondary mechanism is likely to have a greater influence in clinical practice as patients are typically treated for 24 hrs/day for several days with CytoSorb^®^ and the rats were limited to a single 4-hour treatment in this study. The identification of a multitude of proteins from key physiologic pathways modulated by the treatment may potentially aide future studies evaluating treatment responses for a number of indications such as acute liver failure and invasive aspergillosis.

## Material and Methods

### Animal treatment

Eighty-one Sprague Dawley rats (350–450 g) cannulated in the jugular and femoral veins (Taconic Labs) were anesthetized with 1.5% isoflurane and injected with 0.5–1.0 mg/kg of AFB_1_ (1.4 mg/mL solution- 44:56 (v/v) DMF:saline solution) intravenously via the tail vein^48–50^. Hemoperfusion was initiated immediately or after a 30, 90, or 240-minute delay and conducted for 4 hours at a flow rate of 1.5 mL/min from the jugular vein through a device containing 3 mL CS polymer beads or an empty device (Control) and back into the rat through the femoral vein catheter. Humane endpoints for euthanasia were included (i.e., inability to eat and drink for 2 days, loss of >15% body weight or >6 °C drop in body temperature). Heparin was dosed at 200 IU/kg/hour during the hemoperfusion and proper heparinization levels monitored by ACT with a Vetscan i-STAT (Abaxis). Rats were observed for a 7-day period following AFB_1_ exposure with body weight and temperature recorded daily.

### Analytical methods

Blood was collected at specific times prior to, during, and following the hemoperfusion session. Hematological analysis was conducted with a Hemavet analyzer (Drew Scientific). Circulating AFB_1_ and cytokines were measured by ELISA (Helica Biosystems, Santa Ana, CA) and Multi-plex immunoassay (Bio-Plex Th1/Th2 Rat Cytokine Assay Kit; Bio-Rad Laboratories), respectively.

### Histology

At death, livers were preserved in 10% neutral buffered formalin (NBF). Liver tissue sections were stained with haematoxylin and eosin dyes (H&E) and scored for key hepatotoxicity markers: hemorrhage, necrosis, biliary hyperplasia, and inflammation.

### Proteomic analysis

For the 90-min delayed treatment proteomic study: blood samples were collected 30 min (baseline) prior to AFB_1_ dose (1 mg/kg IV) and immediately before and after the 4-hour hemoperfusion. The plasma was frozen and shipped to MS Bioworks (Ann Arbor, MI) for proteomic analysis via liquid chromatography-mass spectrometry (LC-MS). Plasma samples (10 µL) were depleted of the most abundant proteins using Proteome Purify 2 Mouse Serum Protein Immunodepletion Resin (R&D Systems, Catalog no. MIDR002-020) according to manufacturer’s protocol. Depleted samples were buffer exchanged into water on a Corning Spin × 5 kDa molecular weight cut off spin column and quantified by Qubit fluorometry (Life Technologies). 50 μg of each sample was reduced with dithiothreitol, alkylated with iodoacetamide and digested overnight with trypsin (Promega). Each digested sample was processed by solid phase extraction and 3 μg of each digest was analyzed by nano LC-MS/MS with a Waters M-Class NanoAcquity HPLC system interfaced to a ThermoFisher Fusion Lumos mass spectrometer for 3 hrs per sample. Raw label-free quantitation (LFQ) protein intensity values were Log_2_ transformed prior to calculating percent change difference and statistical analysis.

### Statistical analysis

Statistical analyses between individual data points and between survival plots was done with student’s two tailed t-test and log-rank (Mantel-Cox) test, respectively, with alpha = 5.000% (GraphPad Prism).

### Significance statement

Mycotoxins continue to pose a serious threat as biological weapons due to their high systemic toxicity, environmental stability and ready accessibility. With the ongoing and unpredictable threat from terror groups, there is an acute need for an effective medical countermeasure. This study used extracorporeal blood purification with highly porous polymer beads to demonstrate an effective therapeutic method to reduce toxicity and mortality following administration of a lethal dose of aflatoxin in an animal model. Comprehensive evaluation of potential mechanisms indicates the device works through a combination of direct toxin adsorption and modulation of the levels of reactive proteins in the plasma from the aflatoxin-mediated inflammation and tissue damage.

### Ethics statement

All animal work was done in accordance with protocols approved by the Institutional Animal Care and Use Committee at Rutgers University and the US Army Animal Care and Use Review Office.

## Supplementary information

Hemoadsorption Improves Survival of Rats Exposed to an Acutely Lethal Dose of Aflatoxin B1.

## Data Availability

The authors agree to policies on data sharing and materials. The mass spectrometry proteomics data generated during the current study have been deposited to the ProteomeXchange Consortium (http://proteomecentral.proteomexchange.org) via the PRIDE partner repository with the dataset identifier PXD015442.
