# Supplementary material for: Hemoadsorption Improves Survival of Rats Exposed to an Acutely Lethal Dose of Aflatoxin B1
Source: Sci Rep. 2020 Jan 21;10:799. doi: 10.1038/s41598-020-57727-y (PMC6972926; doi:10.1038/s41598-020-57727-y)
Supplement: Supplementary file 1 — Hemoadsorption Improves Survival of Rats Exposed to an Acutely Lethal Dose of Aflatoxin B1. [file 41598_2020_57727_MOESM1_ESM.pdf]

Hemoadsorption Improves Survival of Rats Exposed to an Acutely Lethal Dose of Aflatoxin B_1

Karl-Gustav Ruggeberg, Pamela O'Sullivan, Timothy J Kovacs, Kathryn Dawson1 Vincent J Capponi, Phillip P Chan, Thomas D Golobish, and Maryann C Gruda*

CytoSorbents Medical, Monmouth Junction, United States

Supplementary Information

Circulating Blood Counts

During the immediate treatment study, AFB_1 insult caused an increase in white blood cell (WBC) numbers after 24 hours, from 12 to 18 $x10^3$ cells/µL and 10 to 15 $x10^3$ cells/µL for the Control and CS-treated group, respectively (Table S3). However, only the Control rats had a significant increase in WBCs from baseline after AFB_1 exposure (Paired t-test, $P<0.0005$), indicating that CS treatment is limiting the toxin-mediated increase of circulating WBCs. There was no appreciable change in erythrocyte or platelet levels after 24 hours or significant difference in these parameters between the Control and CS-treated groups (Table S3).

During the 30-minute delayed treatment study, AFB_1 administration caused a sharp drop in circulating WBC numbers over the first 4.5 hours, from 17 $x10^3$ to 6 $x10^3$ cells/µL and 18 $x10^3$ to 8 $x10^3$ cells/µL for the Control and CS-treated group, respectively (Table S3). WBC counts returned to baseline levels within 24 hours of exposure for both the Control and CS-treated groups. Though the WBC count was higher in the CS-treated group compared to the Control rats at 24 hours post-injection, the difference was not significant (t-test $P=0.27$). In addition, the elevated WBC number in the CS-treated rats at 24 hours was not significantly different from that of baseline (Paired t-test $P=0.12$). Platelet numbers increased over 4.5 hours by 30% for both the Control and CS-treated groups. After 24 hours, platelet numbers returned to baseline levels in the CS-treated rats at 448 $x10^3$ cells/µL (Paired t-test $P=0.27$). In contrast, Control group animals experienced a significant 46% drop in platelets below baseline levels (Paired t-test $P<0.04$). There was no appreciable change in erythrocyte number after 24 hours and no significant difference between the Control and CS-treated groups (Table S3).

Similar results were observed in the 90-minute delayed treatment study; AFB_1 dosing caused a rapid decline in WBC numbers over the first 1.5 hours, from 11 $x10^3$ to 7 $x10^3$ cells/µL for both Control and CS-treated groups (Table S3). WBC counts returned to baseline levels within 24 hours of exposure for both the Control and CS-treated groups. Though the WBC count was higher in the Control rats compared to the CS-treated rats at 24 hours post-injection, the difference was not significant (t-test $P=0.76$). In addition, the elevated WBC number in the CS-treated rats at 24 hours was not significantly different from that of baseline (Paired t-test $P=0.20$). Platelet numbers increased over 5.5 hours by 14% and 23% for the Control and CS groups, respectively. After 24 hours, platelet numbers remained at these slightly elevated levels above baseline for both groups. There was minimal change in erythrocyte number after 24 hours, with no significant difference between the Control and CS-treated groups (Table S3).

For the 4-hour delayed treatment study, AFB_1 injection also caused a decline, although to a lesser extent, in circulating WBCs after 4 hours from 11 $x10^3$ cells/µL and 10 $x10^3$ cells/µL to 8 $x10^3$

cells/μL in the Control and CS-treated group, respectively (Table S3). At 8 hours, WBC numbers had rebounded to baseline levels. WBC counts continued to rise through 24 hours in both groups to 13 $x10^3$ cells/μL in the Control group and 17 $x10^3$ cells/μL in the CS group and were significantly different from that of baseline (Paired t-test P=0.034 and P=0.045, respectively). However, there was no significant difference between the two groups at this time (P=0.18). Platelet numbers decreased over 8 hours by 23% and 14% for the Control and CS-treated groups, respectively. After 24 hours, platelet counts continued to decline to 73% and 64% of baseline in the Control and CS-treated group, respectively. There was no significant difference between the two groups at this time (P=0.13). Erythrocyte number changed modestly after 24 hours, with levels in the CS-treated group significantly lower than those of the Control group (7.3 $x10^6$ cells/μL vs. 8.0 $x10^6$ cells/μL) (P=0.01) (Table S3).

Cytokine Response

Pro-inflammatory cytokines were marginally elevated during the hemoperfusion period in the immediate treatment animals, with no significant differences between Control and CS-treated groups (Figure S1 and Table S4). AFB_1 caused a delayed inflammatory effect with substantial increases in pro-inflammatory cytokines IL-1α, IL-1β, IL-2, IL-5, IL-6, IL-12, TNF-α, and IFN-γ d above baseline for both study groups at 24 hours. After 2 days, all eight cytokines were elevated, with the CS-treated group exhibiting higher overall levels of IL-5, IL-6, IL-12, TNF-α, and IFN-γ than the remaining Control animals, despite the single CS treatment occurring immediately after toxin exposure (Figure S1 and Table S4). Anti-inflammatory cytokines IL-4, IL-13, and GM-CSF had a modest increase in the CS-treated rats in the days following the AFB_1 dose, with IL-4 and IL-13 peaking on day 2 at 73 and 101 pg/mL, respectively, and GM-CSF peaking on day 3 at 381 pg/mL (Figure S1 and Table S4). Control rats experienced a similar increase for IL-4. IL-13 and GM-CSF peaked at lower levels in the Control rats, but these differences were not significant. IL-10, also an anti-inflammatory cytokine spiked just 1.5 hours after toxin insult in both CS-treated and Control groups. IL-10 levels in the CS group were blunted in comparison to those of the Control group: 198 pg/mL vs. 550 pg/mL; however, this difference was not significant (t-test P=0.13). The following days, IL-10 levels dropped in the Control group, whereas IL-10 dropped briefly in the CS-treated group, before increasing to a peak level of 351 pg/mL on day 6. By day 7, all cytokine levels in the CS-treated group had declined considerably, with IL-6, IL-13, TNF-α, and IFN-γ returning to baseline levels (Figure S1 and Table S4).

In the 30-minute delayed treatment study, cytokine levels gradually rose in both Control and CS-treated groups for the first 4 days after dosing and were almost identical for IL-1α, IL-2, IL-4, IL-5, IL-10, and IFN-γ (Figure S2 and Table S5). The CS-treated group experienced a smaller spike at 4.5 hours to 204 pg/mL before dropping back down to 120 pg/mL at 1 day. However, this difference was not significant (t-test P=0.33). IL-1β levels increased slightly in Control group rats over the 4 days to 179 pg/mL, whereas CS-treated rats experienced a larger increase to 6345 pg/mL; though this difference was not significant (t-test P=0.31) (Figure S2 and Table S5). TNF-α and IL-6 levels peaked sharply at 1.5 and 4.5 hours, respectively for both Control and CS-treated groups. After day 4, only CS-treated rats survived and continued to experience an increase in cytokines through day 6 before declining on day 7 (Figure S2 and Table S5).

In the 90-minute delayed treatment study, there was a sharp decline in all tested cytokine levels immediately after toxin dosing which persisted through 5.5 hours. Afterwards, cytokines increased on day 1, peaking between day 2 and 3 in both Control and CS groups (Figure S3 and Table S6). There were no differences in cytokine levels between the two groups throughout the 7-day observation period. For GM-CSF and IL-1β, the CS group experienced a more rapid reduction in these cytokine levels after day 4 than observed in Control animals, although these differences were not significant (Figure S3 and Table S6).

During the 4-hour delayed treatment study, all tested cytokines decreased to levels below those of baseline at 4 hours post-toxin dose and began to increase in concentration by 8 hours (Figure S4 and Table S7). Cytokine levels continued to increase on day 1, with the majority peaking on day 2 through 7 in both Control and CS-treated groups. Cytokine levels did not differ significantly between the Control and CS-treated during the 7-day study.

Figures and Tables

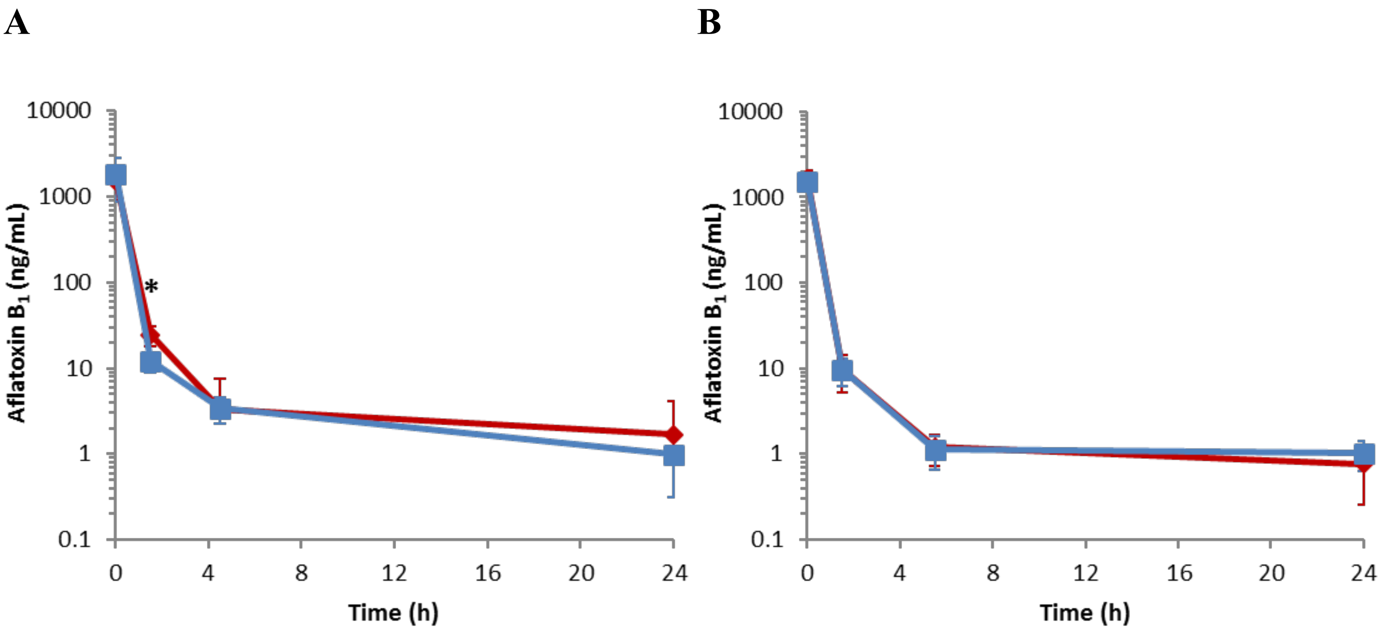

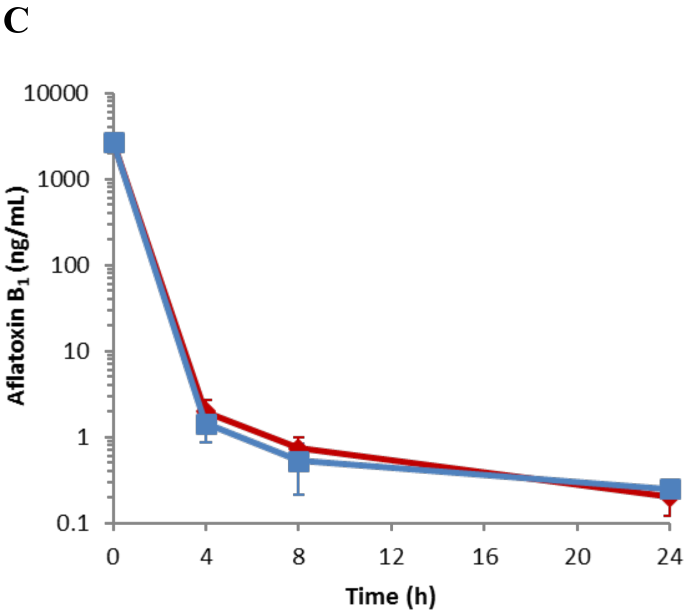

Fig. S1. Concentration of aflatoxin in circulating plasma after dosing. Rats received AFB_1 dose (IV) (0.5mg/kg for 30 and 90-min delay and 1.0mg/kg for 4-h delay) and were treated after a 30-min delay (A), 90-min delay (B), or 4-h delay (C) with an empty control or CS device. Data points represent mean ± SD. n≥8 for both groups, *P<0.05. Concentration of aflatoxin in systemic circulation was determined by competitive ELISA (Helica Biosystems Inc.).

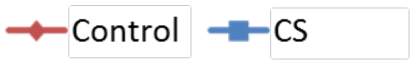

Control	Study	Lethargy	Rough Coat	Chromoda-cryorrhea	Hypothermia
**% Rats with symptoms ≤ 12 hours**	Immediate	25	50	37.5	37.5
	30-Min Delay	37.5	37.5	37.5	12.5
	90-Min Delay	0	16.7	8.3	0
	4-H Delay	25	12.5	37.5	25
**% Rats with symptoms >12 hours**	Immediate	25	12.5	50	0
	30-Min Delay	50	0	25	0
	90-Min Delay	0	8.3	8.3	0
	4-H Delay	12.5	37.5	12.5	0
**% Total Afflicted Rats:**	**Immediate**	**50**	**62.5**	**87.5**	**37.5**
	**30-Min Delay**	**87.5**	**37.5**	**62.5**	**12.5**
	**90-Min Delay**	**0**	**25**	**16.7**	**0**
	**4-H Delay**	**37.5**	**50**	**50**	**25**
**CytoSorb**					
**% Rats with symptoms ≤ 12 hours**	Immediate	10	10	0	10
	30-Min Delay	0	12.5	12.5	12.5
	90-Min Delay	0	8.3	0	0
	4-H Delay	37.5	12.5	25	12.5
**% Rats with symptoms >12 hours**	Immediate	10	10	0	0
	30-Min Delay	25	25	12.5	0
	90-Min Delay	0	0	0	0
	4-H Delay	25	37.5	12.5	0
**% Total Afflicted Rats:**	**Immediate**	**20**	**20**	**0**	**10**
	**30-Min Delay**	**25**	**37.5**	**25**	**12.5**
	**90-Min Delay**	**0**	**8.3**	**0**	**0**
	**4-H Delay**	**62.5**	**50**	**37.5**	**12.5**

Table S1. Physical symptoms observed after AFB_1 dosing and immediate, 30-min, 90-min, or 4-h delayed hemoperfusion. Immediate treatment: n=8 and 10 for Control and CS groups, respectively; 30-min delay: n=8 for both groups; 90-min delay: n=12 and 11 for Control and CS groups, respectively; 4-h delay: n=8 for both groups.

Immediate Treatment		Hemorrhage	Necrosis	Inflammation	Hyperplasia
≤ 2 Days	Control	2.0±1.4	2.0±0	1.0±1.4	0.5±0.7
	CS	2.0	2.0	0	1.0
3-4 Days	Control	1.5±1.3	2.3±1.3	1.8±0.5	2.3±0.5
	CS	2.0±1.4	2.5±0.7	1.0±0	1.5±0.7
5-7 Days	Control	0±0	0±0	1.0±0	1.5±0.7
	CS	0±0	0±0	0.1±0.4	1.9±0.4
**30-Min Delay**					
≤ 2 Days	Control	4.0±0	4.0±0	1.0±0	0±0
	CS	2.0	2.0	1.0	0
3-4 Days	Control	1.5±0.6	2.3±1.0	3.0	2.5±1.0
	CS	2.0	3.0	3.0	0
5-7 Days	Control	-	-	-	-
	CS	0±0	0±0	1.3±0.5	1.7±1.0
**90-Min Delay**					
3-4 Days	Control	0.5±0.6	1.0±0	3.0±0	1.3±0.5
	CS	-	-	-	-
5-7 Days	Control	0±0	0±0	1.6±0.7	1.6±0.9
	CS	0±0	0±0	1.3±0.6	1.4±1.0
**4-H Delay**					
≤ 2 Days	Control	1.0	3.0	2.0	0
	CS	3.5±0.7	3.5±0.7	2.0±0	0±0
3-4 Days	Control	2.2±1.5	2.8±1.1	2.8±0.4	2.0±1.2
	CS	1.3±1.5	1.7±0.6	3.0±0	2.7±0.6
5-7 Days	Control	0±0	1.0±1.4	3.0±0	4.0±0
	CS	0±0	0.3±0.6	2.0±1.0	1.7±1.2

Table S2. Evaluation of effect of CS treatment on AFB_1-induced liver damage. Samples were grouped by survival time and scored on increasing severity from 0 through 4, with 0 representing normal healthy tissue; values are mean ± SD.

Immediate Treatment	Pre		0 hour		1.5 hour		4 hour		24 hour	
	Control	CS	Control	CS	Control	CS	Control	CS	Control	CS
WBC ($x10^3/\mu L$)	12.5±2.0	10.8±2.5	8.7±2.0	8.6±2.3	10.6±2.2	10.7±4.3	10.9±5.4	12.0±4.7	18.4±1.4*	14.6±4.4
Platelets ($x10^3/\mu L$)	777.5±69.5	798.6±112.8	689.0±118.2	649.1±47.1	744.9±61.0	726.9±62.0	760.4±73.5	807.0±69.0	858.8±61.5	790.5±51.0
RBC ($x10^6/\mu L$)	8.2±0.5	8.1±0.4	6.8±0.9	7.2±0.3	7.1±0.4	7.7±0.5	7.0±0.3	7.9±0.5	8.9±0.5	8.1±0.3
**30-Min Delayed Treatment**	**Pre**		**0 hour**		**1.5 hour**		**4.5 hour**		**24 hour**	
	Control	CS	Control	CS	Control	CS	Control	CS	Control	CS
WBC ($x10^3/\mu L$)	17.0±2.7	18.4±3.6	10.2±2.3	8.9±3.6	8.1±3.6	9.1±3.4	6.2±2.1	8.0±3.4	16.2±13.1	24.2±8.5
Platelets ($x10^3/\mu L$)	521.0±105.0	510.0±64.1	697.6±141.8	692.1±128.6	681.5±52.4	736.3±107.2	677.9±107.5	661.5±212.4	292.2±175.7*	447.6±77.4
RBC ($x10^6/\mu L$)	8.6±0.4	8.6±0.4	7.4±0.3	7.7±0.5	7.2±0.3	8.0±0.6	7.2±0.5	7.8±0.6	8.0±1.5	8.4±0.5
**90-Min Delayed Treatment**	**Pre**		**0 hour**		**1.5 hour**		**5.5 hour**		**24 hour**	
	Control	CS	Control	CS	Control	CS	Control	CS	Control	CS
WBC ($x10^3/\mu L$)	12.9±3.6	13.0±3.2	9.7±1.6	9.6±1.7	7.0±1.4	6.4±1.4	6.6±2.8	7.4±3.4	19.2±9.5	18.1±6.6
Platelets ($x10^3/\mu L$)	641.4±89.0	642.7±95.3	694.8±95.2	696.2±78.7	684.6±67.4	679.2±80.2	725.3±93.6	750.4±70.3	758.6±226.7	869.8±254.8
RBC ($x10^6/\mu L$)	8.6±0.6	8.2±0.3	8.0±0.7	7.5±0.5	7.5±0.7	7.2±0.6	7.1±0.4	7.7±0.3	9.2±0.6	8.4±0.5
**4-H Delayed Treatment**	**Pre**		**0 hour**		**4 hour**		**8 hour**		**24 hour**	
	Control	CS	Control	CS	Control	CS	Control	CS	Control	CS
WBC ($x10^3/\mu L$)	10.7±1.1	9.7±1.7	10.65±1.7	9.1±1.5	8.1±1.8	7.6±0.8	10.5±2.3	11.3±2.6	13.2±3.0	17.4±7.9
Platelets ($x10^3/\mu L$)	986.8±184.2	911.4±111.6	537.9±230.7	508.8±255.4	774±68.1	727.1±82.6	761.1±81.5	784.8±64.1	724.4±208.1	585.6±123.5
RBC ($x10^6/\mu L$)	8.4±0.6	7.9±0.3	7.1±0.2	6.8±0.3	7.0±0.3	6.7±0.3	6.6±0.3	7.4±0.4	8.0±0.3	7.3±0.6*

Table S3. Impact of immediate vs. delayed hemoperfusion on blood cell counts after AFB_1 injection. 0.5mg/kg toxin dose given in immediate, 30, and 90-min delayed treatment studies; 1mg/kg in 4 hour delayed treatment study. Toxin was dosed at 0 hours. Values represent mean ± SD. * indicates significant difference from Pre value ($P<0.05$).

IL-1α	Control		CS	
Day	Average	SEM	Average	SEM
-3	17.8	8.7	31.9	14.7
0	7.3	2.1	6.7	1.1
0.063	7.5	1.8	8.1	1.6
0.167	15.8	6.5	12.8	2.2
1	39.7*	7.6	31.5	6.4
2	35.8*	9.0	45.2*	13.3
3	48.0*	12.1	48.0*	7.6
4	21.9		44.9*	7.9
5			47.8*	8.6
6			12.6	7.6
7			22.1	4.8

IL-1β	Control		CS	
Day	Average	SEM	Average	SEM
-3	14.9	6.2	27.6	14.2
0	6.8	1.7	6.7	3.2
0.063	8.2	2.9	4.5	2.6
0.167	41.3	21.1	37.3	14.1
1	99.3	29.3	52.5	8.8
2	65.7	40.7	88.1	30.7
3	191.6	150.4	444.5	354.2
4	113.4		552.6	381.7
5			304.1	278.1
6			78.0	67.0
7			86.4	78.1

IL-2	Control		CS	
Day	Average	SEM	Average	SEM
-3	522.2	308.3	1292.1	606.7
0	0.0	0.0	54.4	37.6
0.063	2.0	2.0	25.5	20.8
0.167	295.4	228.8	65.4	58.1
1	1350.0	290.4	904.9	292.7
2	1282.6	380.4	1564.8	513.8
3	2320.7*	1207.9	2085.8	931.4
4	0.0		2702.1	1621.2
5			4582.2	3640.8
6			4918.0	4391.1
7			2416.4	2229.2

IL-4	Control		CS	
Day	Average	SEM	Average	SEM
-3	22.6	11.4	62.6	30.7
0	2.1	1.0	1.5	0.6
0.063	5.2	2.8	2.5	0.8
0.167	5.6	2.6	2.0	0.6
1	59.6	20.6	43.7	17.6
2	38.3	21.0	73.1*	39.5
3	53.0	40.8	45.9	15.1
4	3.6		37.5	12.9
5			35.5	18.7
6			9.4	9.4
7			17.5	13.1

IL-5	Control		CS	
Day	Average	SEM	Average	SEM
-3	428.9	160.0	704.5	235.0
0	64.6	13.6	91.1	27.0
0.063	92.1	32.1	128.4	31.9
0.167	111.3	49.4	102.6	20.0
1	428.7*	118.2	426.4	103.6
2	303.9	114.7	523.4*	154.9
3	305.1	153.3	503.7*	119.0
4	121.7		405.0	106.8
5			465.7	261.5
6			215.7	141.4
7			254.3	149.2

IL-6	Control		CS	
Day	Average	SEM	Average	SEM
-3	124.4	79.0	409.3	185.9
0	0.1	0.1	1.7	1.4
0.063	0.4	0.4	0.2	0.2
0.167	143.8	138.5	62.3	55.4
1	374.1	102.9	316.9	105.7
2	193.2	108.3	456.0*	203.9
3	289.9	186.2	297.6	81.6
4	0.0		295.8	83.2
5			169.8	63.2
6			64.4	52.9
7			21.0	21.0

IL-10	Control		CS	
Day	Average	SEM	Average	SEM
-3	41.0	11.6	72.0	22.5
0	13.5	6.4	18.6	5.7
0.063	541.2*	203.0	197.9	51.5
0.167	83.1	43.2	54.8	14.2
1	121.1	31.9	74.5	12.1
2	90.2	20.8	120.2	28.2
3	111.0	32.9	114.9	24.0
4	14.0		131.0	49.8
5			247.2	194.7
6			351.4	314.7
7			217.0	176.4

IL-12	Control		CS	
Day	Average	SEM	Average	SEM
-3	156.6	74.8	370.0	191.0
0	19.0	15.9	13.3	6.3
0.063	33.5	28.1	10.9	4.0
0.167	48.8	30.7	3.3	1.2
1	541.8*	190.9	301.8	116.6
2	267.6	137.9	468.7*	251.3
3	319.3	231.9	294.5	124.4
4	0.0		219.4	75.7
5			223.6	136.7
6			52.3	33.6
7			96.5	84.8

IL-13	Control		CS	
Day	Average	SEM	Average	SEM
-3	0.4	0.4	96.9	46.7
0	0.0	0.0	2.8	2.8
0.063	0.0	0.0	0.0	0.0
0.167	7.3	7.3	1.5	1.5
1	5.6	4.8	33.1	19.8
2	8.0	6.8	100.7	57.2
3	35.8	32.6	57.8	20.8
4	0.0		69.7	37.2
5			7.6	7.6
6			8.9	8.9
7			3.6	3.6

GM-CSF	Control		CS	
Day	Average	SEM	Average	SEM
-3	6.7	3.3	17.0	9.3
0	2.7	2.0	4.4	2.2
0.063	4.7	2.9	1.6	0.8
0.167	5.3	2.6	1.0	0.6
1	26.9	5.3	18.0	4.7
2	30.6	20.3	44.8	18.5
3	100.6	78.2	309.7	264.7
4	35.6		381.3	284.2
5			258.4	241.9
6			89.0	89.0
7			105.7	105.7

IFN-γ	Control		CS	
Day	Average	SEM	Average	SEM
-3	54.2	25.9	141.0	68.2
0	0.0	0.0	9.1	5.7
0.063	4.0	4.0	2.1	1.6
0.167	35.8	29.4	6.7	4.5
1	142.3*	38.8	103.0	32.2
2	94.2	37.5	162.8*	73.3
3	69.2	50.1	110.0	23.7
4	14.9		108.7	30.3
5			80.2	26.1
6			44.7	36.0
7			18.4	13.0

TNF-α	Control		CS	
Day	Average	SEM	Average	SEM
-3	154.4	74.5	293.0	139.2
0	0.0	0.0	11.3	11.3
0.063	211.3	135.8	199.8	85.2
0.167	201.4	138.8	215.9	98.6
1	323.9	112.2	383.6	161.9
2	425.5	206.9	728.2	529.3
3	269.5	174.4	451.9	287.3
4	311.8		466.8	270.1
5			191.4	103.4
6			49.6	32.3
7			54.6	43.2

Table S4. Effect of immediate treatment on AFB_1-induced cytokine release. Rats received 0.5mg/kg AFB_1 dose (IV) and were immediately connected to a hemoperfusion circuit containing a Control or a CS device. Values in pg/mL; Mean ± SEM, T0 n=7 and 8 for Control and CS groups, respectively. *Significantly above T0 (P<0.05).

IL-1α	Control		CS	
Day	Average	SEM	Average	SEM
-3	236.8	78.8	217.8	68.2
0	91.8	54.2	86.1	63.8
0.063	9.9	3.7	19.3	5.4
0.187	424.5	195.7	204.4	95.9
1	69.1	20.4	120.4	37.2
2	114.5	34.7	135.9	43.1
3	129.6	50.5	248.1	76.4
4	320.6	193.1	297.0	75.6
5			443.0	78.1
6			601.7*	177.9
7			504.9*	181.3

IL-1β	Control		CS	
Day	Average	SEM	Average	SEM
-3	116.6	38.0	107.4	31.7
0	56.7	34.1	195.0	99.8
0.063	17.4	6.6	134.2	87.7
0.187	529.3*	204.9	654.4	234.0
1	83.9	10.8	322.8	145.7
2	75.7	18.6	553.5	379.8
3	91.1	13.8	1834.1	1430.2
4	179.4	107.6	6345.4	5867.8
5			5087.3	4673.1
6			3501.2	3113.3
7			3019.7	2709.4

IL-2	Control		CS	
Day	Average	SEM	Average	SEM
-3	5637.3	1905.4	5256.0	1764.1
0	2156.6	1349.5	2029.0	1351.8
0.063	68.0	68.0	546.8	211.8
0.187	126.0	62.2	417.3	133.0
1	1204.3	363.0	3333.6	843.7
2	3169.5	732.5	3626.8	1302.9
3	3810.4	940.6	7149.1	2190.0
4	5708.4	2906.4	7991.7	2139.3
5			11943.1*	2629.3
6			14236.1*	3347.3
7			11949.4*	4294.0

IL-4	Control		CS	
Day	Average	SEM	Average	SEM
-3	220.8	58.9	182.6	52.3
0	85.1	47.5	64.1	48.3
0.063	11.4	3.3	17.6	6.0
0.187	15.9	4.6	20.5	3.0
1	36.0	12.4	113.6	29.3
2	111.9	38.9	115.7	42.2
3	117.8	37.9	188.4	63.0
4	246.8	145.5	234.9	69.9
5			383.6*	77.9
6			450.2*	105.2
7			419.9*	146.0

IL-5	Control		CS	
Day	Average	SEM	Average	SEM
-3	604.3	146.0	629.5	150.3
0	312.9	152.1	265.2	124.8
0.063	105.0	14.7	160.9	41.3
0.187	92.0	17.8	140.7	15.6
1	220.5	54.2	460.6	89.8
2	459.9	92.7	486.6	123.0
3	535.3	93.4	715.0	157.5
4	718.8	246.2	801.3	141.6
5			1075.4*	140.4
6			1191.9*	200.8
7			1014.7*	264.3

IL-6	Control		CS	
Day	Average	SEM	Average	SEM
-3	1130.9	351.6	1035.9	308.5
0	347.5	223.1	299.7	240.5
0.063	128.0	116.5	77.6	47.5
0.187	7974.1*	2568.6	8977.4*	5388.2
1	328.7	61.6	608.8	164.7
2	778.9	147.0	527.8	231.7
3	506.6	129.1	872.4	316.2
4	817.3	317.7	963.4	292.3
5			1461.3	273.3
6			1849.4	449.8
7			1638.5	622.1

IL-10	Control		CS	
Day	Average	SEM	Average	SEM
-3	175.7	53.7	151.6	47.0
0	68.4	38.6	59.0	44.1
0.063	255.3*	59.8	231.6	65.2
0.187	100.1	37.1	55.4	14.4
1	82.5	7.5	109.9	23.5
2	114.8	21.2	112.9	37.2
3	113.8	27.5	194.2	59.4
4	203.0	109.4	224.5	58.5
5			338.7*	71.4
6			409.7*	96.4
7			359.8*	122.7

IL-12	Control		CS	
Day	Average	SEM	Average	SEM
-3	626.4	226.6	640.1	208.9
0	288.8	204.8	217.9	167.1
0.063	5.3	3.7	17.6	11.5
0.187	13.3	6.3	42.4	14.3
1	65.5	25.3	351.3	121.3
2	291.7	136.5	299.4	124.6
3	1371.1	1229.4	534.6	222.9
4	1345.0	1132.1	712.0	253.2
5			1283.0*	301.0
6			1352.5*	374.3
7			1249.1*	484.1

IL-13	Control		CS	
Day	Average	SEM	Average	SEM
-3	300.5	117.4	265.0	99.4
0	104.5	68.4	75.7	68.8
0.063	0.0	0.0	27.0	22.2
0.187	0.0	0.0	15.4	15.4
1	29.7	18.7	129.3	48.7
2	138.6	70.6	181.1	110.4
3	163.6	68.1	340.0	130.6
4	136.5	37.4	340.1	117.1
5			548.0	210.5
6			673.3*	198.4
7			578.0*	233.5

GM-CSF	Control		CS	
Day	Average	SEM	Average	SEM
-3	139.3	46.4	130.8	33.7
0	73.9	39.6	306.5	166.3
0.063	0.5	0.5	213.6	165.6
0.187	24.4	9.7	317.3	224.2
1	67.4	11.6	386.4	184.9
2	68.7	34.3	1017.6	817.6
3	94.5	27.5	6323.3	5914.0
4	205.2	129.5	11520.7	10996.6
5			11483.2	11003.9
6			17455.8	17009.4
7			11496.6	11131.1

IFN-γ	Control		CS	
Day	Average	SEM	Average	SEM
-3	518.8	169.0	439.3	134.5
0	179.6	103.4	154.9	100.6
0.063	18.4	7.1	47.8	25.6
0.187	311.7	121.1	154.7	71.4
1	105.1	31.5	254.6	71.8
2	278.5	69.1	260.8	95.3
3	267.5	51.5	420.3	125.6
4	355.2	139.2	472.2	127.1
5			766.8*	192.4
6			921.0*	201.2
7			795.3*	298.0

TNF-α	Control		CS	
Day	Average	SEM	Average	SEM
-3	726.5	249.4	734.2	235.3
0	263.7	183.5	121.0	86.8
0.063	5648.6*	2530.2	4326.2	3142.4
0.187	1377.0	428.6	1188.0	411.1
1	226.8	71.4	273.5	77.0
2	33.2	19.6	176.4	76.9
3	0.0	0.0	281.6	94.7
4	0.0	0.0	317.6	116.2
5			608.4	310.6
6			758.0	229.8
7			666.0	281.9

Table S5. Effect of 30-minute delayed treatment on AFB_1-induced cytokine release. Values in pg/mL; Mean ± SEM, T0 n=8 for both groups. *Significantly above T0 (P<0.05).

IL-1α	Control		CS	
Day	Average	SEM	Average	SEM
-3	350.0	49.3	318.1	41.5
0	209.1	48.6	159.5	45.2
0.063	63.6	24.7	18.9	9.6
0.229	15.3	3.7	10.5	3.0
1	443.4	75.3	361.5	61.2
2	546.6*	102.8	579.3*	68.6
3	565.9*	109.1	445.3*	110.1
4	300.5	70.6	413.4	76.5
5	433.4	91.9	373.8	63.4
6	429.6	108.2	362.2	67.7
7	316.3	104.0	306.6	94.3

IL-1β	Control		CS	
Day	Average	SEM	Average	SEM
-3	165.1	20.9	150.1	22.7
0	100.9	22.0	82.0	22.6
0.063	29.6	11.8	9.4	5.5
0.229	14.7	9.9	9.1	3.2
1	254.4	41.3	205.4	29.0
2	337.1	72.6	331.7*	34.7
3	453.6*	114.3	273.1*	58.5
4	307.3	66.0	284.8*	54.8
5	388.3*	88.4	216.6	37.1
6	363.5	103.4	200.0	33.9
7	277.3	126.0	172.9	57.0

IL-2	Control		CS	
Day	Average	SEM	Average	SEM
-3	8722.2	925.5	8157.4	970.7
0	5490.0	1095.3	4368.1	1159.4
0.063	1635.6	702.5	559.2	333.8
0.229	22.7	13.2	202.5	148.9
1	10645.9	1669.6	9100.6	1285.4
2	12760.0*	2155.0	13596.5*	1362.9
3	13959.4*	2393.4	10743.6*	2693.9
4	7506.3	1945.8	9847.2	1870.9
5	9421.5	1803.6	9681.5	1636.4
6	9981.9	2594.4	8998.3	1758.3
7	6376.4	2037.2	7601.5	2121.0

IL-4	Control		CS	
Day	Average	SEM	Average	SEM
-3	283.7	30.5	281.2	33.1
0	191.8	37.7	149.9	41.0
0.063	52.2	20.9	16.0	8.5
0.229	6.5	1.5	8.7	2.2
1	371.5	58.9	306.9	44.2
2	405.6	72.3	464.9*	44.9
3	436.5*	79.3	328.0	74.8
4	253.2	64.0	323.8	55.7
5	337.9	65.8	312.6	50.1
6	345.8	92.3	303.5	58.1
7	240.7	81.1	243.8	68.3

IL-5	Control		CS	
Day	Average	SEM	Average	SEM
-3	907.3	59.5	869.6	66.3
0	638.7	87.1	560.5	110.2
0.063	279.6	71.3	157.1*	51.4
0.229	105.0*	12.3	137.1*	14.6
1	989.9	110.3	909.6	81.8
2	1108.4*	124.1	1205.1*	83.5
3	1175.5*	129.7	937.3	130.2
4	834.1	98.1	966.2*	113.5
5	1017.5	115.1	949.3	104.3
6	961.4	169.0	904.3	115.7
7	761.3	175.5	770.1	151.2

IL-6	Control		CS	
Day	Average	SEM	Average	SEM
-3	1647.1	156.4	1566.9	159.1
0	1091.9	193.4	857.0	224.9
0.063	364.7	155.4	75.3	75.3
0.229	213.0	202.1	23.2	17.2
1	2003.5	310.4	1701.6	218.1
2	2226.8*	348.4	2408.0*	213.3
3	2140.5	353.4	1653.0	387.0
4	1347.6	371.8	1584.0	302.6
5	1645.0	348.7	1645.9	259.5
6	1951.2	462.6	1586.9	295.5
7	1175.9	406.1	1254.6	288.7

IL-10	Control		CS	
Day	Average	SEM	Average	SEM
-3	286.5	33.3	270.3	29.1
0	178.8	32.8	158.7	40.6
0.063	63.4	23.9	21.2	12.9
0.229	12.1	3.5	10.6	3.3
1	372.7*	58.1	316.8	44.3
2	441.3*	60.4	489.1*	40.1
3	443.7*	69.2	353.7	82.7
4	247.1	56.5	322.3	58.6
5	310.6	54.2	323.9	54.6
6	325.5	75.2	297.5	53.8
7	237.6	73.8	249.8	70.3

IL-12	Control		CS	
Day	Average	SEM	Average	SEM
-3	1028.6	121.5	1018.5	206.2
0	664.0	156.3	546.2	200.5
0.063	162.9	68.5	49.8	33.7
0.229	2.2	1.8	15.7	7.2
1	1426.3	273.4	1210.2	205.4
2	1537.3	373.1	1708.9*	262.1
3	1504.5	346.1	1102.3	248.1
4	711.1	181.3	1150.5	220.0
5	1147.1	296.5	1057.2	194.6
6	1322.0	450.2	1134.9	250.1
7	806.9	262.8	812.3	248.6

IL-13	Control		CS	
Day	Average	SEM	Average	SEM
-3	540.3	66.9	514.9	64.9
0	333.9	78.7	243.7	72.3
0.063	110.6	57.3	22.7	22.7
0.229	2.6	2.6	0.0	0.0
1	685.6	132.7	514.5	82.5
2	833.9*	188.2	796.0*	96.4
3	716.1	149.4	596.7	213.0
4	392.9	144.4	538.9	162.4
5	476.4	132.1	553.5*	113.5
6	570.9	196.7	516.6*	107.4
7	325.7	138.9	368.4	83.4

GM-CSF	Control		CS	
Day	Average	SEM	Average	SEM
-3	178.2	27.3	171.4	28.5
0	120.4	28.3	85.1	28.2
0.063	25.8	12.5	3.7	2.7
0.229	0.3	0.3	3.4	2.4
1	273.2	43.9	220.1	35.4
2	379.5	88.6	371.1*	50.2
3	487.4*	132.8	295.0*	68.3
4	321.9	65.2	304.1*	55.8
5	393.0	80.2	227.2	40.3
6	362.5	109.8	205.3	44.1
7	278.9	121.1	168.9	59.2

IFN-γ	Control		CS	
Day	Average	SEM	Average	SEM
-3	721.4	76.0	712.6	86.1
0	475.4	94.9	331.3	81.6
0.063	154.1	64.9	35.4	26.1
0.229	11.3	5.2	18.6	9.1
1	934.6	149.6	774.6	111.7
2	1075.0*	195.7	1100.7*	106.6
3	968.9	171.8	842.5	257.1
4	591.9	165.8	815.0	203.9
5	765.2	162.3	791.9	151.9
6	815.6	221.7	752.2	163.5
7	558.9	194.5	608.4	162.7

TNF-α	Control		CS	
Day	Average	SEM	Average	SEM
-3	996.8	120.0	1403.3	496.0
0	964.7	323.8	772.8	430.4
0.063	191.0	69.3	80.9	41.2
0.229	83.6	60.8	55.9	27.3
1	1535.3	559.3	1097.3	369.9
2	2038.4	937.0	1577.0	573.4
3	1617.0	696.7	941.2	313.5
4	792.3	477.7	971.8	343.5
5	1076.7	558.1	1003.8	347.7
6	1449.3	960.6	1220.4	571.0
7	548.9	185.9	627.9	226.1

Table S6. Effect of 90-minute delayed treatment on AFB_1-induced cytokine release. Values in pg/mL; Mean ± SEM, T0 n=12 and 11 for Control and CS groups, respectively. *Significantly above T0 ($P<0.05$).

IL-1α	Control		CS	
Day	Average	SEM	Average	SEM
-3	603.4	248.9	564.0	353.5
0	892.0	795.5	691.9	550.4
0.167	31.9	15.1	104.6	45.9
0.33	162.8	77.5	84.0	36.6
1	685.9	307.6	723.9	372.8
2	617.1	342.5	601.5	203.9
3	749.6	420.8	301.4	181.7
4	1004.8	585.2	602.8	291.7
5	627.6	627.6	13.8	13.8
6	0.0	0.0	1675.6	1596.9
7	2485.4	0.0	36.8	36.8

IL-1β	Control		CS	
Day	Average	SEM	Average	SEM
-3	276.6	133.2	318.8	143.9
0	483.7	382.1	350.2	198.8
0.167	41.1	19.0	114.2	50.1
0.33	197.4	91.6	118.5	53.9
1	228.9	84.5	231.7	121.4
2	297.1	142.0	428.6	192.8
3	402.0	127.2	285.5	149.5
4	425.1	139.6	322.7	110.8
5	563.9	209.1	86.9	54.7
6	261.9	0.0	191.1	180.6
7	131.6	0.0	84.9	84.9

IL-2	Control		CS	
Day	Average	SEM	Average	SEM
-3	7093.8	2568.8	5897.6	2658.6
0	4041.6	2089.7	2750.5	1349.9
0.167	791.1	512.3	1806.2	826.6
0.33	2842.4	1419.8	3947.1	1700.5
1	7657.1	1906.7	8412.3	3169.7
2	8747.8	2610.5	8978.0	2348.3
3	8945.2	3032.0	5583.9	2262.0
4	6234.5	3964.3	2764.1	1357.5
5	4545.2	4545.2	801.2	801.2
6	0.0	0.0	14426.1	11162.5
7	19769.1	0.0	8428.6	5498.7

IL-4	Control		CS	
Day	Average	SEM	Average	SEM
-3	309.1	97.3	203.7	98.7
0	121.1	60.3	168.0	82.7
0.167	20.6	8.9	100.1	52.1
0.33	152.9	86.3	79.6	40.8
1	253.0	58.4	188.8	57.5
2	244.7	91.9	235.8	77.8
3	225.4	74.6	223.8	111.3
4	106.4	53.6	167.0	75.8
5	293.9	293.9	54.2	36.6
6	0.0	0.0	205.4	40.2
7	186.9	0.0	89.0	25.7

IL-5	Control		CS	
Day	Average	SEM	Average	SEM
-3	1349.3	337.8	981.5	324.5
0	811.4	222.6	862.0	304.9
0.167	383.6	145.6	677.0	249.8
0.33	840.0	313.9	637.1	177.7
1	1071.6	233.8	914.9	219.5
2	1157.4	330.8	1104.6	281.7
3	1111.2	323.1	1011.0	435.9
4	691.6	290.2	824.2	359.6
5	1351.1	1098.2	366.9	96.1
6	252.9	0.0	787.7	189.1
7	0.0	0.0	566.7	22.7

IL-6	Control		CS	
Day	Average	SEM	Average	SEM
-3	1165.1	428.2	2404.0	1458.9
0	597.3	298.7	748.5	491.4
0.167	64.5	64.5	362.8	222.7
0.33	734.2	401.1	413.8	292.8
1	1041.0	368.5	757.9	216.6
2	4459.3	3870.1	2496.7	1451.8
3	979.2	444.8	1105.3	754.5
4	98.2	98.2	362.7	230.6
5	1168.7	1168.7	146.4	146.4
6	0.0	0.0	819.3	139.5
7	541.4	0.0	332.6	288.2

IL-10	Control		CS	
Day	Average	SEM	Average	SEM
-3	357.7	116.6	298.2	145.7
0	139.0	69.5	160.0	96.8
0.167	5.0	5.0	93.2	48.9
0.33	143.8	82.1	72.7	58.2
1	220.9	75.4	186.7	95.3
2	352.2	224.7	339.5	122.3
3	226.6	103.6	267.2	158.0
4	74.8	49.0	175.1	96.6
5	303.5	303.5	37.2	37.2
6	0.0	0.0	110.9	68.8
7	0.0	0.0	102.2	47.2

IL-12	Control		CS	
Day	Average	SEM	Average	SEM
-3	1534.0	548.3	1104.8	575.0
0	904.9	473.5	963.9	439.5
0.167	50.7	31.8	450.6	243.4
0.33	703.9	418.8	368.0	168.0
1	1092.3	272.3	982.1	333.1
2	974.1	487.7	939.1	306.3
3	978.9	399.2	1097.6	651.2
4	371.0	272.1	819.1	496.4
5	1374.9	1340.7	100.6	50.5
6	41.6	0.0	2237.4	1963.3
7	2441.8	0.0	214.8	85.9

IL-13	Control		CS	
Day	Average	SEM	Average	SEM
-3	232.5	110.4	222.1	130.2
0	491.2	266.4	196.8	163.7
0.167	73.6	73.6	229.5	132.2
0.33	295.2	196.4	232.4	130.9
1	282.5	154.6	209.8	94.1
2	277.8	168.9	305.3	188.8
3	285.6	155.1	296.4	227.5
4	0.0	0.0	28.6	28.6
5	195.2	195.2	0.0	0.0
6	0.0	0.0	40.6	40.6
7	0.0	0.0	25.2	25.2

GM-CSF	Control		CS	
Day	Average	SEM	Average	SEM
-3	650.2	402.1	627.8	446.0
0	579.6	495.2	571.1	450.2
0.167	3.9	3.9	76.1	43.3
0.33	148.6	86.2	502.0	420.4
1	651.6	442.1	1112.7	580.6
2	1130.2	464.3	1135.3	515.3
3	1482.8	630.1	1353.4	574.5
4	2117.8	989.6	1039.2	578.4
5	2399.1	1806	1301.3	1173.0
6	4113.2	0.0	2149.1	2083.7
7	4075.7	0.0	2014.0	1992.6

IFN-γ	Control		CS	
Day	Average	SEM	Average	SEM
-3	582.2	201.7	492.3	211.2
0	373.7	160.1	479.7	274.8
0.167	60.5	39.9	250.1	125.6
0.33	345.7	183.6	213.1	112.8
1	445.7	154.6	303.2	112.8
2	448.2	231.0	457.1	198.0
3	475.8	264.0	577.0	426.7
4	90.3	62.6	280.1	146.9
5	523.7	523.7	80.1	80.1
6	0.0	0.0	323.2	74.6
7	115.2	0.0	194.8	194.8

TNF-α	Control		CS	
Day	Average	SEM	Average	SEM
-3	996.2	354.4	927.6	423.5
0	833.0	337.6	709.5	285.6
0.167	39.9	39.9	369.1	194.4
0.33	632.7	340.2	648.8	340.7
1	959.6	369.8	620.5	278.4
2	829.6	325.6	889.8	353.6
3	647.7	374.9	749.8	421.1
4	164.9	164.9	634.2	286.2
5	502.5	502.5	269.2	269.2
6	0.0	0.0	494.6	55.7
7	264.7	0.0	147.3	147.3

Table S7. Effect of 4-hour delayed treatment on AFB_1-induced cytokine release. Values in pg/mL; Mean ± SEM, T0 n=8 for both groups.

Functional Class	Protein ID	Protein Name	% Change
Heat Shock/ Chaperone	P06761	78 kDa glucose-regulated protein	1.76
Metabolism/ Nutrient Transport	F1M6Z1;Q7TMA5	Apolipoprotein B-100; Apolipoprotein B-48	-0.60
	A0A0A0MY39;Q9QYJ4	ATP-binding cassette sub-family B member 9	1.58
	G3V8B1;Q8R2H5	Phosphatidylinositol-glycan-specific phospholipase D	-1.11
	A0A0G2K6Y4;D3ZW66	Small G protein signaling modulator 2	2.55
pH and Fluid Balance	B0BNN3	Carbonic anhydrase 1	-7.18
	P27139	Carbonic anhydrase 2	-5.73
Blood Coagulation/ Hemostasis	F1LST1	Fibronectin 1	1.48
	Q5EBC0;D3ZFC6	Inter-alpha-trypsin inhibitor heavy chain H4	-0.70
	P01048	T-kininogen 1	1.22
	M0R5R0;P53813	Vitamin K-dependent protein S	-2.22
Complement Activation Pathway	Q5M891;Q63514	C4b-binding protein alpha chain	-1.19
	D3ZWD6	Complement C8 alpha chain	0.65
	Q6MG73	Complement component 2	1.39
	A0A096P6L9;A0A1B0GWS5	Complement component 5	0.61
	F1M983;G3V9R2	Complement factor H	0.40
	A0A0G2K135;Q9WUW3	Complement factor I	0.39
	P08661;A0A140UHW8	Mannose-binding protein C	1.13
Hemoglobin Component	P01946;G3V8R3	Hemoglobin subunit alpha-1	-5.86
	A0A0G2JSV6	Hemoglobin subunit alpha-2	-12.45
	P02091	Hemoglobin subunit beta	-5.88
	A0A0G2JTW9	Hemoglobin subunit beta-1	-6.94
	P11517	Hemoglobin subunit beta-2	-6.50
Serine Protease/ Endopeptidase Inhibitor	G3V9J1	Adhesion molecule with Ig like domain 3	-0.59
	Q5EBA7;ENSBTAP00000023055	HGF activator	-0.40
	P04916	Retinol-binding protein 4	0.63
	F1LR92;Q63556	Serine protease inhibitor A3M	0.53
	A0A0H2UHI5;A0A0G2KB85	Serine protease inhibitor A3N	0.85
	Q80ZA3;CON__Q95121	Serine protease inhibitor f1	-0.82
Inflammation/ Apoptosis	G3V836;P05371	Clusterin	-0.56
	G3V6K6	Receptor protein-tyrosine kinase	-0.54
ROS Detoxification	Q08420	Extracellular superoxide dismutase [Cu-Zn]	1.24
	A0A0G2JSH9;P35704	Peroxiredoxin-2	-10.47

Table S8. AFB_1-induced changes in plasma proteins at 90 minutes. Proteins identified by LC-MS/MS analysis significantly changed ($P<0.05$) in abundance from baseline (-30 min) to 90 minutes after AFB_1 administration (1mg/kg). Change is calculated as % change from baseline (n=16).

Functional Class	Protein ID	Protein Name	% Change
Heat Shock/ Chaperone	P06761	78 kDa glucose-regulated protein	5.45
Metabolism/ Nutrient Transport	G3V9R9;P36953	Afamin	-0.33
	P24090;F1LM19	Alpha-2-HS-glycoprotein	0.40
	P04638	Apolipoprotein A-II;Proapolipoprotein A-II	-2.87
	F1M6Z1;Q7TMA5	Apolipoprotein B-100	-3.83
	P19939;A0A0G2K9R5	Apolipoprotein C-I	-4.88
	A0A0G2K8Q1;P06759	Apolipoprotein C-III	-3.25
	P55797	Apolipoprotein C-IV	-4.09
	A0A0A0MY39;Q9QYJ4	ATP-binding cassette sub-family B member 9	4.16
	Q6IRK9	Carboxypeptidase Q	1.40
	Q68FS4	Cytosol aminopeptidase	4.38
	A0A0G2JYL5	Dynein Axonemal Heavy Chain 2	-1.13
	P51886;CON__Q05443	Lumican	1.54
	P59996	Proprotein convertase subtilisin/kexin type 9	-2.85
	O55004	Ribonuclease 4	1.96
	P02770	Serum albumin	-2.85
	A0A0H2UHH2;P23680	Serum amyloid P-component	1.34
	P02767	Transthyretin	0.76
pH and Fluid Balance	Q5U329;F8WFT7;P23562	Anion exchange protein;Band 3 anion transport protein	-4.68
	B0BNN3	Carbonic anhydrase 1	-5.59
Blood Coagulation/ Hemostasis	Q5I0M1;P26644	Beta-2-glycoprotein 1	0.65
	Q9EQV9;CON__Q2KIG3	Carboxypeptidase B2	1.68
	P16296	Coagulation factor IX	1.66
	A0A0G2K4I9;Q6TUF8	Coagulation factor XI	1.62
	A0A0H2UI19;D3ZTE0	Coagulation factor XII	0.57
	B1H260;F6Q1N1	Coagulation factor XIII B chain	1.35
	A0A0G2K9Y5;A0A0G2K3G0	Histidine-rich glycoprotein	0.65
	A2VD04;F1M8H8	Hyaluronan-binding protein 2	-2.44
	Q5EBC0;D3ZFC6	Inter-alpha-trypsin inhibitor heavy chain H4	-1.11
	Q5M878;Q7TMC3	Serum amyloid A protein	-1.49
	F7FMY6;P31394	Vitamin K-dependent protein C	1.94
	M0R5R0;P53813	Vitamin K-dependent protein S	-3.19
	G3V8K8	Vitamin K-dependent protein Z	-1.42
Complement Activation Pathway	G3V7L3	Complement C1s subcomponent	1.42
	D3ZWD6	Complement C8 alpha chain	0.75
	Q6MG73	Complement component 2	5.43
	A0A096P6L9;A0A1B0GWS5	Complement component 5	1.33
	Q811M5	Complement component C6	0.99
	P55314	Complement component C8 beta chain	0.86

	D3ZPI8	Complement component C8 gamma chain	1.46
	F1M983;G3V9R2	Complement factor H	0.81
	Q5I0M3;Q7TP43	Complement Factor H Related 1	1.92
	P08661;A0A140UHW8	Mannose-binding protein C	2.29
Hemoglobin Component	P01946;G3V8R3	Hemoglobin subunit alpha-1	-3.67
	A0A0G2JSV6	Hemoglobin subunit alpha-2	-10.34
	P02091	Hemoglobin subunit beta	-2.89
	A0A0G2JTW9	Hemoglobin subunit beta-1	-4.87
	P11517	Hemoglobin subunit beta-2	-4.80
Serine Protease/ Endopeptidase Inhibitor	P14046	Alpha-1-inhibitor 3	-1.04
	P31211	Corticosteroid-binding globulin	1.53
	P14841	Cystatin-C	1.55
	A0A0G2K8K3;Q64268	Heparin cofactor 2	1.79
	Q5EBA7; ENSBTAP00000023055	HGF activator	-0.73
	Q5M8C3	Serine protease inhibitor 4 (Kallistatin)	1.49
	P05545	Serine protease inhibitor A3K	1.00
	P05544;A0A0G2JXK5	Serine protease inhibitor A3L	1.47
	F1LR92;Q63556	Serine protease inhibitor A3M	1.15
	A0A0H2UHI5;A0A0G2KB85	Serine protease inhibitor A3N	2.43
	A0A0G2JYK0;F1LM05	Serine protease inhibitor A3N	1.43
	Q5M7T5;CON__P41361	Serine protease inhibitor clade C	1.74
Inflammation/ Apoptosis	P15473;A0A0G2K4Q9	Insulin-like growth factor-binding protein 3	2.28
	P21744	Insulin-like growth factor-binding protein 4	-9.19
	F1M9B9;D1M8S3	Interleukin-1 receptor accessory protein	-0.72
	F7FMS0	Macrophage-stimulating 1	1.13
	Q3KR94;Q7TQ11	Vitronectin	-4.04
Proteasome Component	Q6P9V6;P34064	Proteasome subunit alpha type-5	3.11
	P18420	Proteasome subunit alpha type-1	2.63
Structural	P60711;A0A0G2K3K2	Actin, cytoplasmic 1	10.34
Unknown Function	D4A6E3;A0A0G2JUW7	LOC100911833-Hypothetical	0.40
	F1LVK0;A0A096MJT6	RGD1307100-Hypothetical	-4.12
	A0A0G2K896;E9PST1;Q6TUG7	RGD1310507-Hypothetical	0.63

Table S9. AFB_1-induced changes in plasma proteins 5.5 hours after dosing. Proteins significantly changed ($P<0.05$) in abundance from baseline (-30 min) to 5.5 hours after AFB_1 administration (1mg/kg) in Control rats (n=8). Change is calculated as % change from baseline.

Functional Class	Protein ID	Protein Name	% Change
Heat Shock/ Chaperone	P63018;D4A4S3	Heat shock cognate 71 kDa protein	2.52
Metabolism/ Nutrient Transport	P24090;F1LM19	Alpha-2-HS-glycoprotein	-3.05
	E9PSQ1;E9PSI7	Alpha-amylase	-1.51
	M0R5J4;P04764	Alpha-enolase	3.98
	P01015	Angiotensinogen	-0.69
	P04638	Apolipoprotein A-II	-0.97
	P02651;A0A0G2JVX7	Apolipoprotein A-IV	-2.63
	A0A0G2K151;P02650	Apolipoprotein E	1.42
	Q5M890	Apolipoprotein N	2.37
	P07824	Arginase-1	2.43
	P07335	Creatine kinase B-type	-2.85
	P05065;Q6AY07	Fructose-bisphosphate aldolase A	3.20
	P04642;H9N9H4	L-lactate dehydrogenase A chain	3.79
	P51886;CON__Q05443	Lumican	-0.96
	O88989	Malate dehydrogenase, cytoplasmic	3.13
	D3ZTX4	Maltase-glucoamylase	2.61
	G3V8B1;Q8R2H5	Phosphatidylinositol-glycan-specific phospholipase D	0.95
	Q5XI38;A0A0G2K014	Plastin-2	3.55
	P59996	Proprotein convertase subtilisin/kexin type 9	2.72
	P85973	Purine nucleoside phosphorylase	2.50
	P11980;A0A0H2UI07	Pyruvate kinase PKM	3.27
	A0A0H2UHH2;P23680	Serum amyloid P-component	1.43
	F7FAY5	Similar to alpha-fetoprotein	-1.56
	G3V826;P50137	Transketolase	2.58
	P02767	Transthyretin	-1.36
	A0A0G2K542;Q4V8I9	UTP-glucose-1-phosphate uridylyltransferase	3.70
	D4A183	Vascular non-inflammatory molecule 3	1.05
	F1LQS6;P22985	Xanthine dehydrogenase/oxidase	1.88
	Q3B8R6;Q63678	Zinc-alpha-2-glycoprotein	-2.48
pH and Fluid Balance	B0BNN3	Carbonic anhydrase 1	4.33
	P27139	Carbonic anhydrase 2	5.69
Blood Coagulation/ Hemostasis	Q63041	Alpha-1-macroglobulin (45kDa Subunit)	1.20
	P06238	Alpha-2-macroglobulin	1.72
	Q5I0M1;P26644	Beta-2-glycoprotein 1	-1.62
	Q9EQV9;CON__Q2KIG3	Carboxypeptidase B2	-0.88
	P16296	Coagulation factor IX	-2.09
	A0A0H2UHR6;Q63207	Coagulation factor X	-1.65
	A0A0H2UI19;D3ZTE0	Coagulation factor XII	-2.19
	G3V811	Coagulation factor XIII A chain	1.15
	Q7TQ70;P06399	Fibrinogen alpha chain	0.93
	P14480	Fibrinogen beta chain	1.04
	P02680	Fibrinogen gamma chain	1.11
	D3ZQU7	Glycoprotein Ib-alpha	2.87

	A0A0G2K9Y5;A0A0G2K3G0	Histidine-rich glycoprotein	-1.30
	B2RYM3	Inter-alpha-trypsin inhibitor heavy chain 1	0.92
	D3ZFH5;CON__Q9TRI1	Inter-alpha-trypsin inhibitor heavy chain 2	0.94
	Q01177;Q7TP84	Plasminogen	-1.06
	G3V843;P18292	Prothrombin	-1.11
	P12346;A0A0G2QC06	Serotransferrin	-0.65
	Q5M878;Q7TMC3	Serum amyloid A protein	0.74
	F7FMY6;P31394	Vitamin K-dependent protein C	-1.92
Complement Activation Pathway	Q9EQV8;CON__Q2KJ83	Carboxypeptidase N catalytic chain	0.94
	F1LQT4	Carboxypeptidase N subunit 2	1.42
	A0A0G2JW12;M0RB00	Complement C4-A	1.05
	Q6MG90	Complement C4-A	0.93
	Q811M5	Complement component C6	-2.23
	A0A0G2K7X7	Complement component C7	-1.30
	F7F389;Q62930	Complement component C9	-0.95
	A0A0U1RRP9;Q7TP05	Complement factor B	-0.85
	F1M983;G3V9R2	Complement factor H	0.66
	A0A0G2K135;Q9WUW3	Complement factor I	-0.72
	Q5M8B4;Q9WTS8	Ficolin-1	3.23
Hemoglobin Component	P01946;G3V8R3	Hemoglobin subunit alpha-1	3.34
	A0A0G2JSV6	Hemoglobin subunit alpha-2	6.89
	P02091	Hemoglobin subunit beta	3.65
	A0A0G2JTW9	Hemoglobin subunit beta-1	4.75
	P11517	Hemoglobin subunit beta-2	5.65
Serine Protease/ Endopeptidase Inhibitor	G3V9J1	Adhesion molecule with Ig like domain 3	1.17
	A0A0G2JY31;A0A0G2JZ73	Alpha-1-antiproteinase	-0.95
	A0A0G2K8K3;Q64268	Heparin cofactor 2	-1.80
	P04916	Retinol-binding protein 4	-3.03
	Q5M8C3	Serine protease inhibitor 4 (Kallistatin)	-1.43
	P05545	Serine protease inhibitor A3K	-1.08
	P05544;A0A0G2JXK5	Serine protease inhibitor A3L	-1.21
	F1LR92;Q63556	Serine protease inhibitor A3M	-1.62
	A0A0H2UHI5;A0A0G2KB85	Serine protease inhibitor A3N	-1.56
	A0A0G2JYK0;F1LM05	Serine protease inhibitor A3N	-1.29
	F7FHF3	Serine protease inhibitor clade f	-2.01
Inflammation/ Apoptosis	Q99J86	Attractin	1.85
	P07151	Beta-2-microglobulin	-4.30
	D4AA52;A0A0G2K926	C3+PZP-like alpha-2-macroglobulin domain-containing protein8	0.58
	G3V836;P05371	Clusterin	-1.09
	D3ZJF8	Fc fragment of IgG binding protein	2.81
	Q6IRS6;Q9QX79	Fetuin-B	-1.66
	F7FMS0	Macrophage-stimulating 1	-1.46
	P29534;A0A0G2K127	Vascular cell adhesion protein 1	4.19
	Q68FY4;P04276	Vitamin D-binding protein	-1.07
	Q3KR94;Q7TQ11	Vitronectin	-1.47
Proteasome Component	Q6P9V6;P34064	Proteasome subunit alpha type-5	1.80
	P17220	Proteasome subunit alpha type-2	3.88
	P18422	Proteasome subunit alpha type-3	2.33

	Q6PDW4;P18421	Proteasome subunit beta type-1	2.63
	P40307	Proteasome subunit beta type-2	2.59
	P40112;D3Z8J0	Proteasome subunit beta type-3	2.70
ROS Detoxification	Q08420	Extracellular superoxide dismutase [Cu-Zn]	-1.99
	M0RAM5;P04041	Glutathione peroxidase 1	5.92
	A0A0G2K531;P23764	Glutathione peroxidase 3	1.29
	A0A0G2JSH9;P35704	Peroxiredoxin-2	4.44
	P25236;A0A0G2JU99	Selenoprotein P	-2.63
	P10960;F7EPE0	Sulfated glycoprotein 1	1.67
Structural/ Misc. Proteins	A0A0G2K1C0;Q4V7C7	Actin-related protein 3	3.90
	M0RDM4;Q6I8Q6	Histone H2A	4.95
	M0R4L7;G3V9C7	Histone H2B	3.39
	P62804	Histone H4; Osteogenic growth peptide	2.69
	Q63610;A0A140TAF0	Tropomyosin alpha-3 chain	2.47
Unknown Function	A0A0G2JVP4;F1LM30	A0A0G2JVP4	3.19
	D4A6E3;A0A0G2JUW7	LOC100911833-Hypothetical	0.74
	A0A0G2K896;E9PST1	RGD1310507-Hypothetical	-0.64

Table S10. Effect of CS treatment on AFB_1 (1mg/kg)-induced changes in circulating protein abundance following 4-hr hemoperfusion (n=8). All listed proteins were significantly impacted ($P<0.05$). Change in levels is calculated as fold change in abundance (CS/Control at 5.5h post toxin dose).

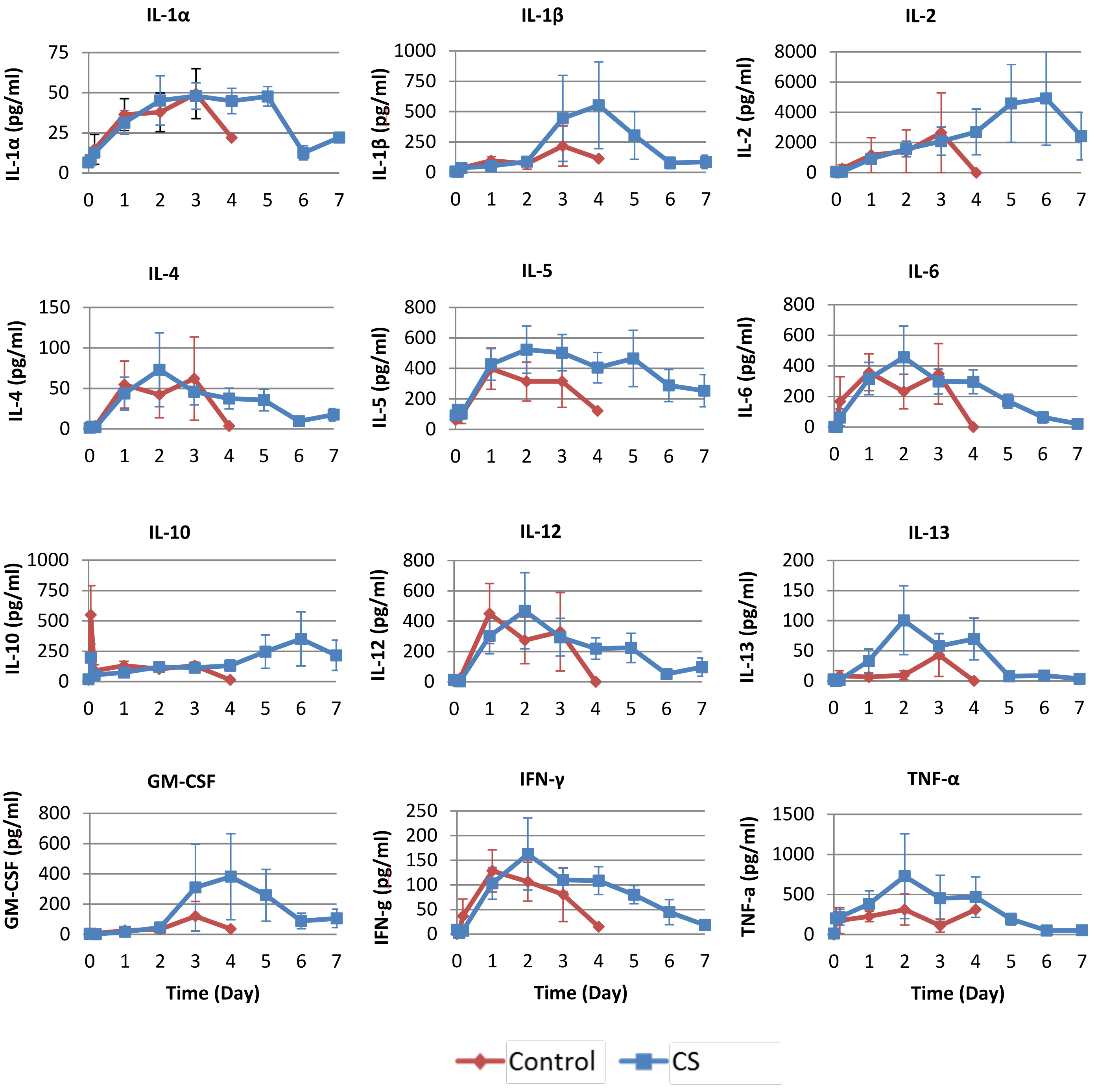

Figure S2. Effect of immediate hemoperfusion on AFB_1-induced cytokine release. Mean ± SEM.

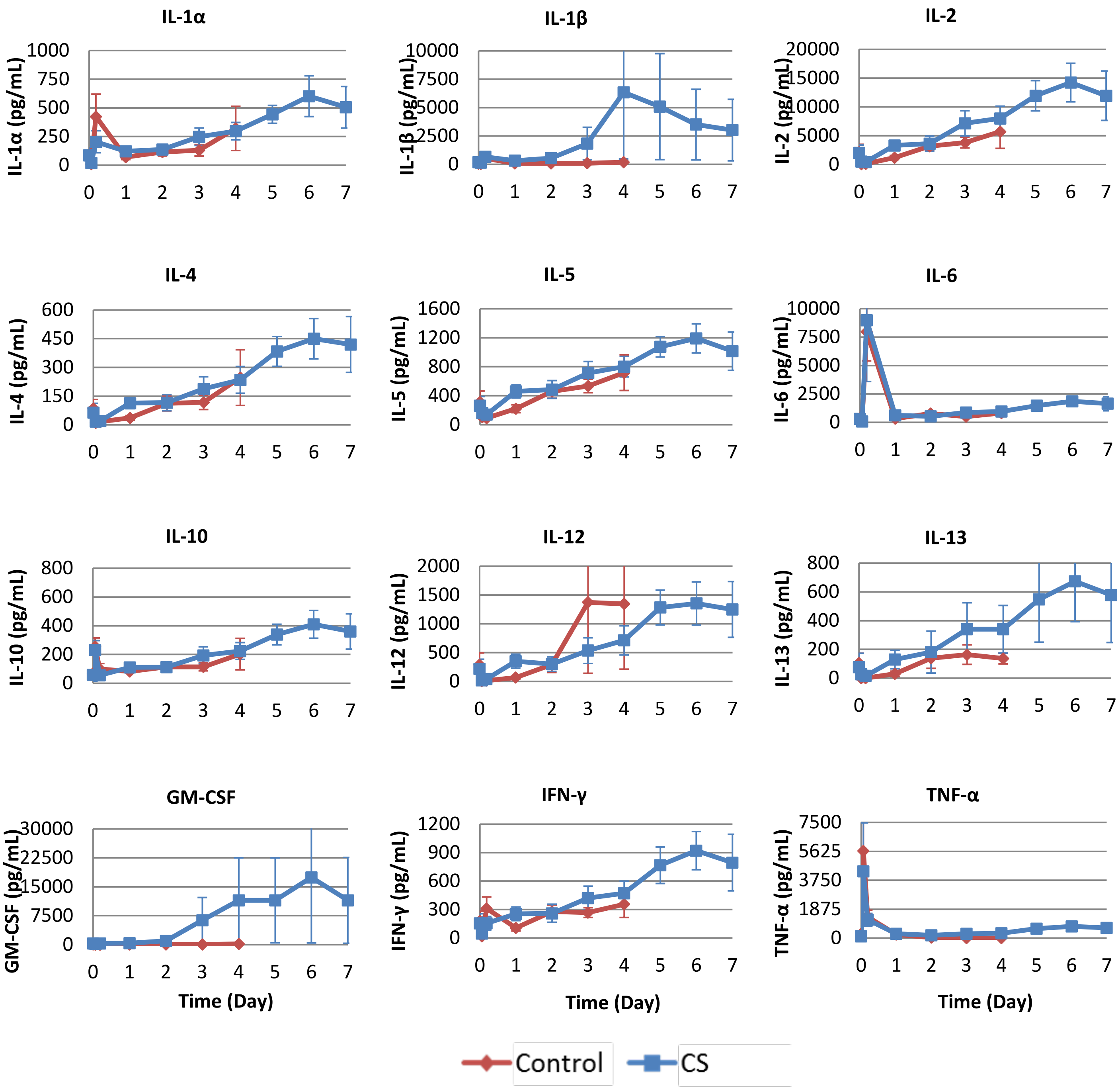

Figure S3. Effect of 30-minute delayed treatment on AFB_1-induced cytokine release. Mean ± SEM.

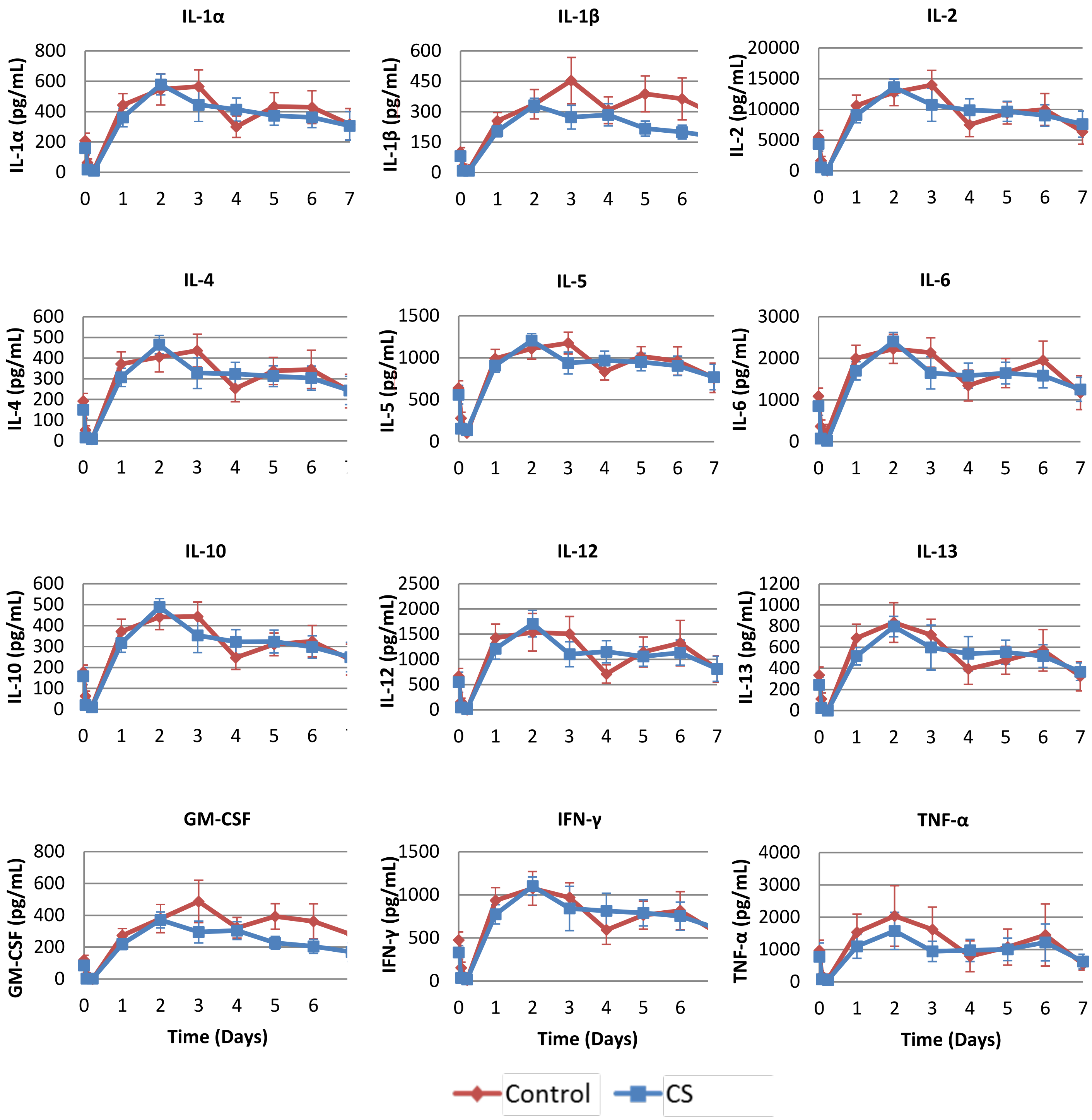

Figure S4. Effect of 90-minute delayed treatment on AFB_1-induced cytokine release. Mean ± SEM.

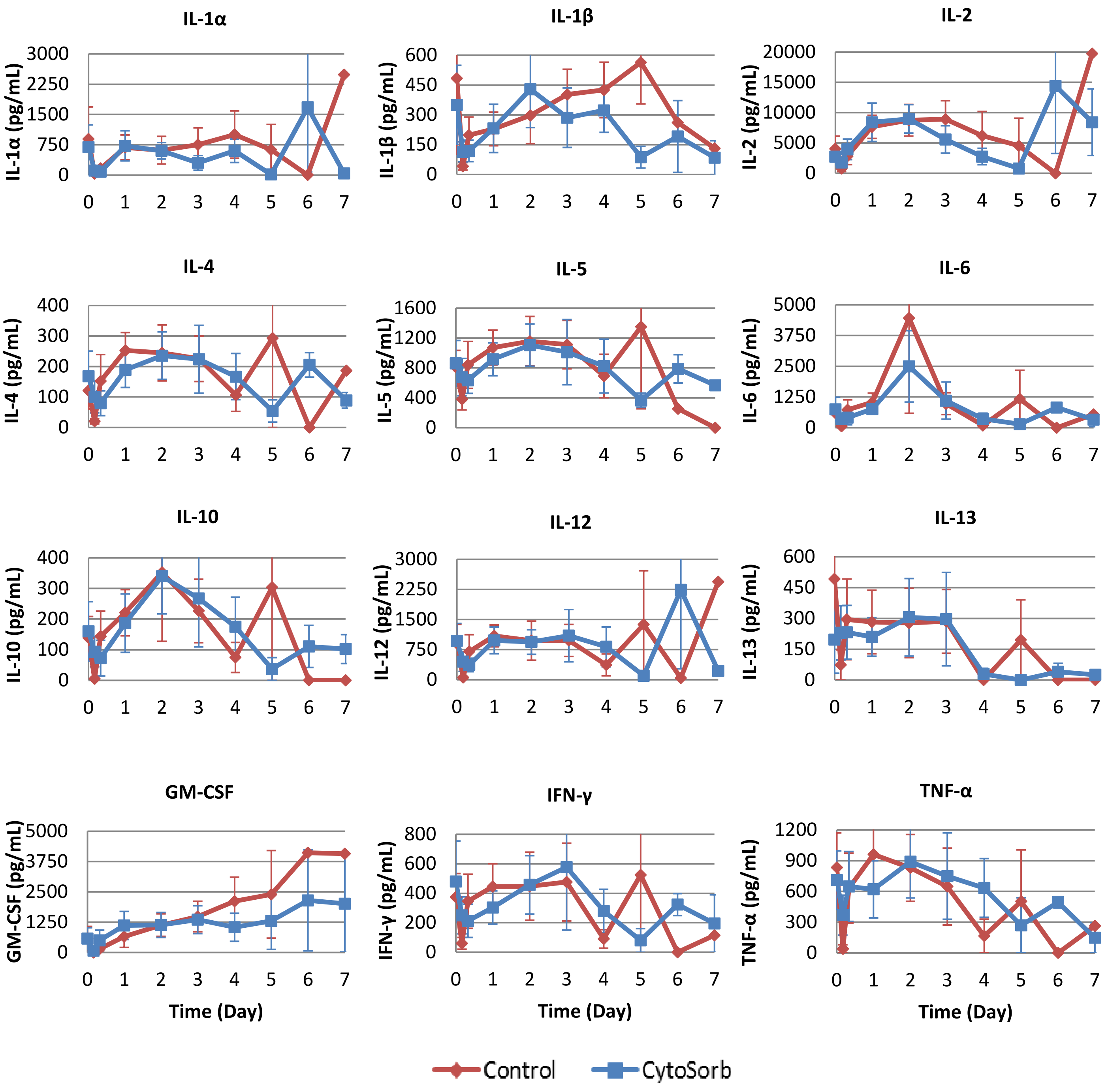

Figure S5. Effect of 4-hour delayed treatment on AFB_1-induced cytokine release. Mean ± SEM.
